# Machine Learning-Guided Prediction of Formulation Performance in Inhalable Ciprofloxacin–Bile Acid Dispersions with Antimicrobial and Toxicity Evaluation

**DOI:** 10.1021/acs.molpharmaceut.5c00663

**Published:** 2025-10-11

**Authors:** Tareq Zeyad Bahjat, Twana Mohammed M. Ways, Sadat Abdulla Aziz, Aram Ismael Ibrahim, Deon Danto, Veneece Ghattas, Goran Mohammed Raouf, Glyn Barrett, Dana Khdr Sabir, Pyman Mohamed Mohamedsalih, Hisham Al-Obaidi

**Affiliations:** † School of Pharmacy, 6816 University of Reading, Reading RG6 6AD, U.K.; ‡ Department of Pharmaceutics, College of Pharmacy, 275719 University of Sulaimani, Sulaymaniyah, Kurdistan Region 46001, Iraq; § Department of Anaesthesia, College of Health Sciences, Cihan University-Sulaimaniya, Sulaymaniyah, Kurdistan Region 46001, Iraq; ∥ Department of Basic Sciences, College of Vet. Medicine, University of Sulaimani, Sulaymaniyah, Kurdistan Region 46001, Iraq; ⊥ Basic Medical Sciences, College of Medicine, University of Sulaimani, Sulaymaniyah, Kurdistan Region 46001, Iraq; # School of Biological Sciences, University of Reading, Reading RG6 6AD, U.K.; ¶ Department of Medical Laboratory Sciences, College of Science, 601125 Charmo University, Chamchamal, Kurdistan Region 46023, Iraq

**Keywords:** ciprofloxacin, dry powder inhaler (DPI), solid dispersions, bile acids, spray drying, lung deposition, antimicrobial activity, machine learning

## Abstract

Ciprofloxacin (CFX) is a potent antibiotic for respiratory infections, but its poor solubility and high crystallinity limit its effectiveness in dry powder inhaler (DPI) delivery. Although soluble forms such as CFX hydrochloride are available, their rapid dissolution may lead to systemic absorption, undermining localized lung targeting. To address this, we developed solid dispersions of CFX with primary bile acids, namely, cholic acid (CA) and chenodeoxycholic acid (CDA), using spray drying and ball milling to enhance solubility in a controlled manner while maintaining deposition in the lungs. Differential scanning calorimetry showed glass-transition temperature (*T*_g_) values were elevated for both bile acids, with CA dispersions showing slightly higher absolute values (114.16–131.77 °C vs 109.13–120.67 °C). However, Fourier transform infrared and dissolution data indicated that CDA formed stronger directional hydrogen bonding with CFX. X-ray diffraction confirmed partially amorphous dispersions with minimal residual crystallinity. Solubility enhancement was observed for both bile acids, showing slightly higher values with CA dispersions. Aerodynamic assessments using an Andersen cascade impactor revealed improved lung deposition with CFX–CDA, with a higher fine particle fraction (FPF: 30.81%) and lower mass median aerodynamic diameter (MMAD: 5.89 μm) compared to CFX–CA (FPF: 26.93%, MMAD: 6.19 μm). The emitted dose was highest in CDA with nearly 5 mg compared to CA dispersions (∼3 mg). In vitro antimicrobial studies showed that dispersions maintained comparable antimicrobial activity to pure CFX, while in vivo toxicology in rats indicated mild, dose-dependent hepatic changes. CDA formulations showed AST elevation at a low dose and ALP increase at a high dose, consistent with the known hepatic effects of this bile acid, while CA formulations were broadly comparable to pure CFX. Machine learning algorithms, including tree-based models and neural networks, were used to predict the formulation performance and identify critical variables. Feature selection was achieved using recursive elimination, and permutation analysis showed that the bile acid type, inlet temperature, and molar ratio were the most influential predictors of solubility and lung deposition. Models such as gradient boosting and elastic net showed a high predictive accuracy (*R* ^2^ > 0.85). Overall, this study highlights the potential of primary bile acid-based DPI formulations as effective inhalable antibiotic therapies.

## Graphical abstract

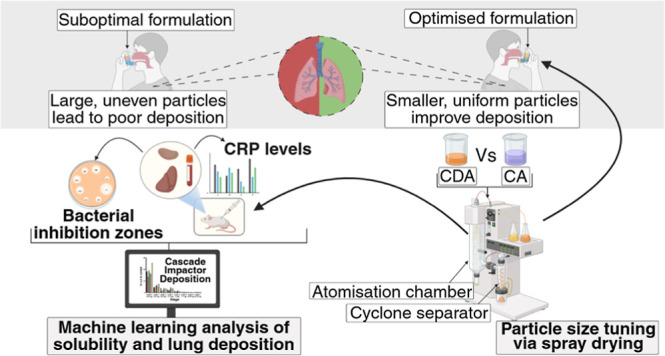

## 1 Introduction

Inhalation drug delivery systems, including nebulizers, metered-dose inhalers, and dry powder inhalers (DPIs), offer noninvasive treatment options with minimal systemic side effects.^1^ Among these, DPIs stand out for their efficiency but face challenges such as particle agglomeration, which can hinder drug delivery to the lower airways.^2^ To overcome this, carrier molecules like lactose are commonly used to enhance drug flowability and dispersion.^3^ However, this strategy has limitations, such as low active pharmaceutical ingredient (API)-to-carrier ratios and the risk of powder deposition in the upper airways, necessitating careful consideration of detachment forces during inhalation.^4^ These challenges are especially critical in the treatment of respiratory tract infections caused by bacteria such as *Klebsiella pneumoniae*. Such infections often lead to biofilm formation, which makes bacteria resistant to natural defenses of the host and therapeutic agents.^5^

Ciprofloxacin (CFX), a second-generation fluorinated quinolone antibiotic, is effective against respiratory infections, particularly those caused by Gram-negative bacteria like *K. pneumoniae*.^6^ Despite its efficacy, CFX’s clinical use is limited by adverse effects such as nephrotoxicity and neurotoxicity.^7^ To address this, innovative inhalable delivery methods are being developed to reduce the required dosage and associated side effects.^8^ However, CFX’s poor water solubility (∼0.09 mg/mL at 37 °C) poses a significant hurdle. This is due to its zwitterionic nature, resulting from a negatively charged carboxylic acid and a positively charged secondary amine, which creates high crystal lattice energy and low solubility in aqueous media.^9^ While soluble salts of CFX, such as CFX hydrochloride, exist, they tend to dissolve rapidly and may be systemically absorbed, reducing local lung retention.

To address solubility and stability challenges, spray-dried solid dispersions have emerged as an innovative strategy. These systems involve binary or multicomponent mixtures that stabilize poorly water-soluble drugs and improve dissolution profiles.^10^ Depending on the coformers used, solid dispersions can be classified as drug-excipient or drug–drug systems. Drug-excipient systems employ coformers like urea, sugars, and carboxylic acids to enhance physical stability and dissolution rates.^11^ Drug–drug systems, on the other hand, stabilize two pharmacologically active components in a single formulation, offering synergistic effects and high drug loading.^12^ These systems enhance dissolution through a “spring effect,” where rapid supersaturation occurs upon administration, and maintain this state via a “parachute effect,” which delays recrystallization and supports sustained absorption.^13^ Precipitation inhibitors are often added to further extend this stability and absorption period.^14^

Primary bile acids, specifically cholic acid (CA) and chenodeoxycholic acid (CDA), play an essential role in overcoming solubility challenges and enhancing drug performance. These amphipathic molecules, synthesized from cholesterol in the liver, act as emulsifying agents in lipid digestion and absorption.^15^ Their dual hydrophilic and hydrophobic nature enables them to form micelles, reducing surface tension and improving the solubility of hydrophobic compounds.^16^ Beyond this, bile acids serve as natural biosurfactants and can help disrupt bacterial membranes which can boost the efficacy of antimicrobial agents.^17^ Here, in this work, we introduce a novel approach by using physiological molecules to augment the effect of CFX. We show that incorporating bile acids into spray-dried dispersions improves the solubility and can enhance antimicrobial activity. In addition, spray-dried formulations containing bile acids offer a promising approach for inhalable drug delivery, reducing crystallinity and optimizing drug performance.^18^ We also evaluated DPI performance using parameters like mass median aerodynamic diameter (MMAD) and fine particle fraction (FPF).^19^

In this study, solid dispersions were prepared by spray drying and mechanical milling and optimized for DPIs aimed at delivering CFX with CA or CDA to the lungs. We recently showed the use of machine learning (ML) techniques can provide significant insights into engineering particles for nose-to-brain targeting.^20^ We use the same approach in this study in which ML is applied to systematically capture complex interactions between formulations and process variables to predict and explain variations in solubility, coarse fine particle fraction (CFP), crystallinity, and fine particle fraction (FPF). A data set comprising 26 unique formulations, each described by 14 input features (e.g., bile acid type, molar ratio, inlet temperature, *T*_g_, and solubility parameters), was used to predict 12 outcome variables including solubility, crystallinity, aerodynamic properties, and yield. Fourteen regression algorithms—including linear models (ridge and elastic net), tree-based methods (random forest, gradient boosting, and extra trees), kernel models (support vector regression), and a neural network (MLP)—were evaluated. To mitigate overfitting risks associated with small sample sizes, 5-fold cross-validation was employed, and only models with *R* ^2^ > 0 and RMSE within twice the target standard deviation were retained. Model performance was assessed by using *R* ^2^, RMSE, and statistical significance tests (paired *t* tests). Feature importance was extracted using permutation importance and SHAP (Shapley Additive Explanations). Notably, inlet temperature, molar ratio, and bile acid type consistently emerged as dominant predictors—findings that align with experimental observations. Together, this integrative approach highlights the potential of bile acid-based spray-dried systems for enhanced DPI formulations and demonstrates the utility of ML not only in predictive modeling but also in extracting interpretable design rules for rational formulation.

## 2 Experimental Section

### 2.1 Materials

CFX powder with a purity of ≥98.0% (HPLC) was procured from Sigma-Aldrich (Dorset, UK). CA powder from bovine and/or ovine, ≥98% (CAS 81-25-4), and CDA were purchased from Sigma-Aldrich (Dorset, UK). HPLC-grade acetonitrile, gradient grade (≥99.9% purity), was acquired from Fisher-Scientific Limited (Leicestershire, UK). Glacial acetic acid was purchased from Sigma-Aldrich (Dorset, UK). Ethanol absolute (≥99.8%) was sourced from Sigma-Aldrich (Dorset, UK). Ultrapure water (HPLC gradient grade) was obtained from Fisher Scientific UK. Unless specified otherwise, all other chemicals were analytical grade.

### 2.2 Preparation of Solid Dispersions by Spray Drying

Spray drying experiments were executed using a B-290 spray dryer (Büchi Labortechnik AG, Switzerland) in a closed-loop configuration with a nitrogen atomizing gas and a nitrogen drying atmosphere. The aspirator was set to 100%, generating a chamber pressure of −100 mbar, while the atomizing gas flow valve was adjusted to a flow rate of 660 L/h. A three-fluid nozzle with a 0.5 mm diameter was employed at a pump rate of 10 mL/min. Two feedstocks were simultaneously pumped through distinct nozzle channels using separate synchronized pumps, with synchronization achieved by measuring the time needed to pump a specific volume of solvents.

Each batch underwent experiments at inlet temperatures ranging from 120 to 160 °C, starting when the outlet temperature stabilized at 60 °C, and it was crucial to maintain the outlet temperature below the glass-transition temperatures of the components.

Feedstocks were prepared in 1:1, 1:2, and 2:1 molar ratios as two solutions, each with a volume of 50 mL of solvent. Initially, CFX and bile acids were separately dissolved in aqueous and organic solvents, respectively. Subsequently, the organic (bile acid) and aqueous (CFX) phases were directed into different channels of the nozzle and internal and external feedstocks. The process yield was calculated as a percentage representing the solids obtained from the spray dryer sample collection point. Reference samples containing only CFX and bile acid solutions were also generated under identical spray drying conditions.

### 2.3 Preparation of Solid Dispersions by Ball Milling (Mechanochemical Activation)

As a comparative approach to the spray drying technique, milling was employed for the preparation of solid dispersions. Pure CFX and bile acid powders, in equimolar ratios of 1:1, 1:2, and 2:1, with a total weight of 1 g, were subjected to milling using the high-energy planetary ball mill Retsch MM 500 NANO (Retsch GmbH, Germany). The mixtures were placed in a stainless-steel grinding jar with a capacity of 50 mL, along with stainless-steel balls (5 mm in diameter) at a weight ratio of 20:1 (ball to powder). The milling oscillation, set at a frequency of 30 HZ per second, was sustained for a continuous mixing duration of 30 min.

### 2.4 Thermogravimetric Analysis

Thermal degradation and potential moisture loss from the formulations were assessed through TGA using a TA Q50 instrument (New Castle, DE, USA). The analysis was conducted by weighing around 5–10 mg of the samples, which were then heated from room temperature to 600 °C at a rate of 10 °C min^–1^ under a nitrogen atmosphere. The data were analyzed using TA Universal Analysis software to determine the moisture content and onset of events.

### 2.5 Differential Scanning Calorimetry

Differential scanning calorimetry (DSC Q2000, TA Instruments, UK) was utilized for thermal analysis of the solid dispersions. The samples were positioned in a crimped T zero aluminum pan and hermetically sealed. To mitigate concerns associated with pressure accumulation during the heating process, a minute pinhole was created in the lid by using a fine needle. A standard thermogram was acquired by allowing the samples to be heated up to 220 °C at a rate of 10 °C min^–1^. Nitrogen gas environment at a flow rate of 50 mL min^–1^ was used to purge all of the samples. An empty, hermetically sealed reference pan with a pierced lid served as a reference. The resulting thermograms were analyzed using Universal Analysis 2000 software (TA Instruments, UK).

### 2.6 Fourier Transform Infrared Spectroscopy

Fourier transform infrared (FTIR) data of spray-dried and milled formulations were collected using a PerkinElmer 100 FTIR spectrometer equipped with a diamond attenuated total reflectance accessory (Shelton, Connecticut, USA). Transmission was recorded with 16 scans’ average, employing a resolution of 4 cm^–1^ and a transmission mode ranging from 700 to 4000 cm^–1^ frequency. Prior to each sample analysis, the stage and crystal were cleaned by using ethanol. The obtained FTIR spectra were compared with reference materials, and data interpretation was executed by using the SpectraGryph 1.2 spectroscopy software.

### 2.7 X-ray Powder Diffraction

The powder X-ray diffraction (XRPD) patterns of CFX, bile acids, and spray-dried and milled samples were obtained using a Bruker D8 Advance X-ray diffractometer (Bruker AXS GmbH, Germany). The X-ray beam, sourced from copper, was directed through a theta-diffractometer equipped with a Lynx eye position-sensitive detector for precise measurements. The Bruker D8 ADVANCE instrument was operated with a 40 kV generator voltage and a 40 mA generator current. Analysis was performed using DFFRAC plus XRD Commander software (Bruker AXS GmbH, Germany) within a 2θ range spanning 5–45°, with a step size of 0.02° and a duration time of 1.33 s per step.

### 2.8 Scanning Electron Microscopy

The particle morphology of the prepared powders was examined by employing a scanning electron microscope (SEM), and images of the specimens were acquired using an FEI Quanta 600F scanning electron microscope (Oregon, USA). The specimens were mounted on aluminum stubs using glued carbon tabs and subsequently sputter-coated with gold for 3 min at 30 mA, utilizing an Emitech K550 system. Imaging was conducted under high vacuum conditions and subsequently enhanced through adjustments in brightness, contrast, and astigmatism correction to evaluate the particle size and morphology.

### 2.9 In Vitro Dissolution and Solubility Analysis

The solubility of the samples was measured in phosphate buffer solution (PBS, 0.1 M, pH = 6.8). Each sample, comprising 6 mg, was added to 1 mL of PBS in triplicate, and the amount was sufficient to prevent complete dissolution. Three sets of samples were mechanically mixed in a rotatory mixer for 1 and 24 h at 25 ± 1 °C. At the specified time point, the samples were centrifuged at 13,000 rpm for 5 min, and the supernatant was collected and filtered with a 0.2 μm syringe filter (Ministart). The CFX concentration was determined using UV–vis spectroscopy at 278 nm, following the generation of a calibration curve (*r* ^2^ > 0.99).

### 2.10 In Vitro Aerodynamic Performance—Anderson Cascade Impaction

Powder deposition in the lungs and the particle size of the DPI were analyzed by the Andersen cascade impactor (ACI), with a flow control rate meter (COPLEY Scientific, Colwick, UK). To ensure the 4 kPa pressure and the required pump flow of 60 L min^–1^ before each run, the cascade suction (inhalation) time was set to 4 s. The spray-dried sample (30 mg) for each run was loaded into a hydroxypropyl methylcellulose hard capsule (size 3) and in an RS01 DPI device (Berry Global, USA). The capsule was pierced by the DPI for loading the dose before inserting the inhaler into the induction port of the mouthpiece to start the run. ACI is of 8 stages with an aerodynamic diameter cutoff of the particle size of each of the 0–7 stages of ACI: 8.6, 6.5, 4.4, 3.3, 2.0, 1.1, 0.54, and 0.25 μm, respectively.^21^ After each run (repeated three times), each stage of the ACI was washed using 10 mL of 3% acetic acid aqueous: absolute ethanol (1:1), except for the CFX spray-dried sample, which was washed with 10 mL of 3% acetic acid. The concentration of each component of the solution was measured using HPLC, as explained below. Coarse particle fraction (CPF), emitted dose (ED), extra-fine particle fraction (EFPF), fine particle dose (FPD), fine particle fraction (FPF), geometric standard deviation (GSD), and MMAD were calculated using an Excel-based macro. The macro applies log-normal transformations to derive MMAD and GSD, adjusts aerodynamic diameter for flow rate variations, and determines FPF and CPF based on the mass deposition profile across impactor stages. The macro-enabled Excel file used for these calculations is provided in the Supporting Information. Full details on how calculations were made are included in the Supporting Information.

### 2.11 Quantification by High-Performance Liquid Chromatography (HPLC)

The concentration of CFX was quantified using an HPLC system (Agilent Technologies series 1200 HPLC system comprising an autosampler, binary pump, column oven, and DAD detector) equipped with a Kinetex-C18 column (150 mm × 4.6 mm, internal diameter 5 μm, Phenomenex, UK). Isocratic elution was conducted at 35 °C, with the mobile phase consisting of 3% acetic acid/acetonitrile, 75:25, %v/v. The injection volume was 20 μL, and the flow rate was 0.8 mL/min, with a run time of 15 min at λ_max_ 278 nm. Calibration curves demonstrated linearity (*R* ^2^ = 0.999), with a retention time of 1.5 min of CFX. The presented data represent mean ± SD of three measurements.

### 2.12 Minimum Inhibitory Concentration

The minimum inhibitory concentration (MIC) against a clinical isolate of *Klebsiella pneumonia* was determined by using the broth microdilution technique following CLSI and EUCAST guidelines. The powdered samples were diluted with deionized water in seven progressive concentrations, ranging from 0.064 to 0.001 mg/mL CFX. Bacterial growth identification was facilitated by the addition of resazurin dye, exhibiting a color change in the presence of viable bacteria. The isolate was grown in Muller–Hinton broth (MHB) to the optical density (OD) of ∼0.8 at λ_max_ 600 nm. Additionally, 5 separate 2-fold dilutions of each formulation were prepared from the respective stock solution. Each well of the 96-well plate contained 20 μL of prediluted bacterial culture (OD = 0.2) derived from an overnight culture, 180 μL of media, and 20 μL each of the different concentrations of each compound, except for the positive control where the number of tested formulations was replaced with media alone. The effect of each formulation was analyzed by checking the bacterial growth represented as the OD at 600 nm after 18 h incubation at 30 °C.

### 2.13 In Vivo Toxicological Investigation

#### 2.13.1 Preparation of the Samples

Two different CFX bile acid formulations (CFX–CA and CFX–CDAL) were dissolved in 3% acetic acid and 10% ethanol to prepare dispersions of the formulations. One milliliter of each of these dispersions was equivalent to a CFX dose of 20 mg/kg for low dose and 60 mg/kg for high dose.

#### 2.13.2 Toxicological Profiles

To investigate the toxicological profiles of the prepared formulations, 21 male Sprague–Dawley rats were used. The rats were acclimatized by housing in a standard environment (12 h light–dark cycle, 25 ± 1 °C) and fed a standard rats’ diet with free access to water. The rats that weighed around 220–250 g were randomly divided into six treatment groups and one control group (3 rats per group) and were orally treated with the corresponding doses (equivalent to a CFX dose of 20 mg/kg for low dose and 60 mg/kg for high dose) for 14 consecutive days. The rats in the control group were treated with the vehicle with no drug.

#### 2.13.3 Blood Sample Collection

At the end of the experiment (day 15), the rats were euthanized using intraperitoneal injection of a xylazine/ketamine mixture (xylazine 35 mg/kg + ketamine 320 mg/kg). About 8 mL of the blood was collected from each rat through a heart puncture. Around 6 mL of the blood was kept in clot activator tubes to separate serum by centrifugation (3600 rpm for 10 min) to be used for biochemical tests, and the rest of the collected blood (about 2 mL) was kept with ethylenediaminetetra-acetic acid and directly used for complete blood count.

#### 2.13.4 Biochemical Tests

The levels of serum urea, creatinine, total protein (TP), albumin (ALB), alanine aminotransferase (ALT), aspartate aminotransferase (AST), total bilirubin (BILT3), C-reactive protein (CRP4), alkaline phosphatase (ALP), and globulin (GLB) were measured using standard diagnostic kits and a Cobas e601 instrument (Roche, Germany). Lipid profiles, including triglyceride, total cholesterol, low-density lipoprotein (LDL), very low high-density lipoprotein, and high-density lipoprotein (HDL), were measured using a biochemical analyzer (Accent 200, Cormy, Poland).

#### 2.13.5 Hematological Assessment

Total red blood cells (RBC), hematocrit (HCT), hemoglobin (Hgb), mean corpuscular volume (MCV), mean corpuscular hemoglobin, mean corpuscular hemoglobin concentration, red cell distribution width—coefficient of variation, and red cell distribution width—standard deviation, together with total and differential white blood cells (WBC) and platelets were measured using an automatic hematology analyzer (Mythic 22, Orphee, Switzerland).

#### 2.13.6 Histopathological Examination

After being euthanized, the organs including liver, spleen, and kidney and the skeletal muscles were aseptically collected from each rat, washed with normal saline, and kept in 10% formaldehyde for 48 h. Then, the histopathological sections were prepared by passing the tissue sections through various steps of dehydration, paraffin-embedding, rehydration, slicing (3 μm), and staining with Harris hematoxylin and eosin (H&E) stain. Finally, the prepared slides were checked under an ordinary microscope, and photographs from each section were taken.

#### 2.13.7 Ethical Approval of In Vivo Studies

In vivo studies were conducted according to the relevant ARRIVE guidelines and regulations and were approved by the Ethics and Research Registration Committee of the College of Pharmacy (Approval No. PH 154-25), University of Sulaimani, Kurdistan Region, Iraq.

### 2.14 ML Workflow

#### 2.14.1 Input Parameters for Model Development

The data set was constructed by compiling experimental results from solubility tests, solid-state characterization, aerodynamic assessments, and process conditions. Each formulation entry was described using a combination of physicochemical properties and formulation/process variables.

The physicochemical descriptors included: weighted molar volume, Hildebrand solubility parameters (δ), Hansen solubility components (δ*D*, δ*P*, and δ*H*), weighted enthalpy of vaporization, and glass-transition temperature (*T*_g_). These parameters were computed using group contribution methods and literature data (Supporting Information Table X).

The formulation and process parameters included:

Bile acid type (CDA or CA), CFX-to-bile acid molar ratio (e.g., 1:1 and 1:2), inlet temperature (120–160 °C), nozzle configuration (internal or external), and milling status. Categorical inputs were one-hot-encoded prior to model training.

The target variables predicted by the models included:

CPF, MMAD, GSD, FPD, ED, EFPF, solubility metrics (mean, min, max, SD), crystallinity percentage, and process yield. Each target was modeled independently.

All of the data were consolidated and curated to ensure completeness. Missing values were imputed by using median substitution. The final data set captured 14 predictor variables and 12 target outcomes across N formulations.

#### 2.14.2 Model Development and Evaluation

Model development was performed using Python (v3.10) with scikit-learn and XGBoost libraries. A total of 14 regression models were tested, including both linear (linear regression, ridge, lasso, and elastic net) and nonlinear algorithms (random forest, extra trees, gradient boosting, XGBoost, LightGBM, support vector regression, k-nearest neighbors, AdaBoost, Gaussian process regression, and multilayer perceptron). Each model was independently trained for every target variable by using the same input features. Data were randomly split into 80% training and 20% test sets. Hyperparameter tuning was performed using 5-fold cross-validation on the training set with grid search where applicable. The primary evaluation metrics were coefficient of determination (*R* ^2^) and root-mean-squared error (RMSE). For comparative analysis, the mean absolute error (MAE) and mean-squared error (MSE) were also computed. All results are reported on the held-out test set, unless otherwise stated. Each model’s performance was recorded for all target variables, and the top three models per target were selected based on *R* ^2^ and RMSE rankings. To assess whether performance differences between models were statistically significant, a paired Student’s *t* test was applied to the error values. Final model selection was based on the best-performing algorithm per target, and those models were carried forward for feature importance analysis.

#### 2.14.3 Interaction Analysis and Feature Importance

To identify which formulation and process parameters had the greatest influence on each outcome, model interpretability analyses were conducted on the best-performing models per target. Permutation importance was first calculated across all features using the test set, measuring the change in prediction error when each feature’s values were randomly shuffled. This allowed for ranking of variable influence while maintaining model-agnostic consistency. In addition, recursive feature elimination (RFE) was applied using a random forest model to filter out features with minimal contribution, ensuring model sparsity without compromising predictive performance. To further enhance interpretability, SHAP (Shapley Additive Explanations) values were computed for tree-based models using the SHAP Python library. SHAP values quantify the marginal contribution of each feature to individual predictions, offering insights into both global and local model behaviors. Summary plots and violin plots were generated to visualize the distribution and stability of feature effects across different models. Features such as inlet temperature, molar ratio, enthalpy of vaporization, and solubility parameters consistently ranked highest across CPF, solubility, crystallinity, and FPD targets. In contrast, categorical variables such as the nozzle type and bile acid identity showed target-specific influence, particularly on solubility and aerodynamic outcomes. This combination of permutation ranking, RFE filtering, and SHAP-based interpretation ensured robust identification of the most influential parameters, aligning the computational findings with mechanistic formulation insights.

### 2.15 Statistical Analysis

Unless otherwise specified, all the data were computed and presented as mean values ± standard deviation based on three replicate measurements. Paired Student’s *t* test and one-way analysis of variance (ANOVA) with post hoc Tukey test were performed using Minitab (Minitab Inc., Version 20, State College, PA, USA) to assess the differences between groups. Statistical significance was considered at *P* < 0.05 or <0.01.

## 3 Results and Discussion

### 3.1 Analysis of Critical Processing Parameters

Table 1 shows the enthalpy of vaporization, molar volume, and Hildebrand solubility parameters for CFX, CDA, and CA. The data for enthalpy of vaporization and molar volume were obtained from the Royal Society of Chemistry’s ChemSpider database. Hildebrand solubility parameter data were calculated using the group contribution method (see Supporting Information). The enthalpy of vaporization reflects the energy required to break intermolecular forces and transition from liquid to vapor. CFX has the highest melting point (255 °C), indicating strong solid-phase intermolecular interactions, likely due to hydrogen bonding, dipole–dipole interactions, and π-stacking from its aromatic system. However, its enthalpy of vaporization is the lowest (136.19 kJ/mol), suggesting that intermolecular forces in the liquid phase are relatively weaker, allowing for easier vaporization. This discrepancy between its solid-state and liquid-state behavior highlights the importance of considering both melting point and enthalpy of vaporization when evaluating intermolecular forces.

**Table 1:** Enthalpy of Vaporization (kJ/mol), Molar Volume, and Hildebrand Solubility Parameter of CFX and Bile Acids^a^

compound	enthalpy of vaporization (kJ/mol)	molar volume (cm^3^/mol)	Hildebrand solubility parameter (MPa^1/2^)
CFX	136.19	226.8	24.88
CDA	160.14	347.9	21.46
CA	197.67	344.8	23.94

a The data for enthalpy of vaporization and molar volume were obtained from the Royal Society of Chemistry’s ChemSpider database. Hildebrand solubility parameter data were calculated using the group contribution method.

Bile acids, in contrast, exhibit lower melting points (CDA: 168 °C, CA: 198 °C) but higher enthalpies of vaporization (160.14 and 197.67 kJ/mol, respectively). This indicates that while their solid-state packing might not be as strong as CFX’s, their liquid-phase intermolecular interactions are stronger, primarily due to extensive hydrogen bonding and van der Waals interactions. The difference between the two bile acids can be attributed to the additional hydroxyl group in CA, which increases the hydrogen bonding and overall cohesion.

The Hildebrand solubility parameter (δ) provides further insight into the solubility behavior. CFX, with the highest δ value (24.88 MPa^1/2^), is the most polar, suggesting strong hydrogen bonding and dipole–dipole interactions with polar solvents such as water and alcohols. The bile acids exhibit slightly lower solubility parameters (CA: 23.94 MPa^1/2^, CDA: 21.46 MPa^1/2^), reflecting their amphiphilic nature, where hydroxyl and carboxyl groups contribute to polarity, while the steroidal core provides a hydrophobic domain. Since solubility parameters reflect cohesive energy density, similar values indicate favorable molecular interactions, which are crucial for forming solid dispersions. The slight difference in solubility parameters suggests that CA is more compatible with CFX than CDA, likely due to its additional hydroxyl groups enhancing hydrogen bonding with the drug molecule. Nonetheless, as seen later, performance metrics such as faster dissolution and enhanced lung deposition suggest CDA offers more functionally beneficial interactions.

### 3.2 Preparation of the Solid Dispersions and Estimating Drug Contents

#### 3.2.1 Morphology and Residual Moisture Analysis of Formed Dispersions

To assess the levels of residual organic solvent in the samples postspray drying, thermogravimetric analysis (TGA) was conducted. Minimum noticeable weight loss was observed in the 70–120 °C region. This temperature range aligns with the boiling points of ethanol, water, and acetic acid at 78 °C, 100 °C, and 117 °C, respectively. SEM images of the spray-dried formulations are presented in Figure 1. CFX–CA particles displayed a spherical morphology with slightly corrugated surfaces. The images indicated the presence of some elongated particles, possibly attributed to the ethanol percentage in the spray-drying feedstock. CFX–CA particles exhibited heavily folded walls, resulting in a disc-like shape. This shape could be attributed to the rapid evaporation of ethanol and CA precipitation at the droplet surface, forming a spherical wall that collapsed after water evaporation. SEM images of binary formulations revealed particles with similar shapes—spherical with smooth surfaces and occasional wall depressions. The residual moisture content for all spray-dried particles is below 5% (data not shown). CFX–CDA displays the highest moisture content at 4.88 ± 0.08%, a value significantly higher than all other formulations (*p* < 0.05). Following this, CFX–CA exhibits the second-highest moisture content, followed by 2:1, with no statistically significant difference between the moisture content of these two formulations. Notably, formulations 1:1 and 1:2, characterized by a higher CA content than 2:1, demonstrate significantly lower moisture content compared to the other formulations (*p* < 0.05).

**Figure 1:**
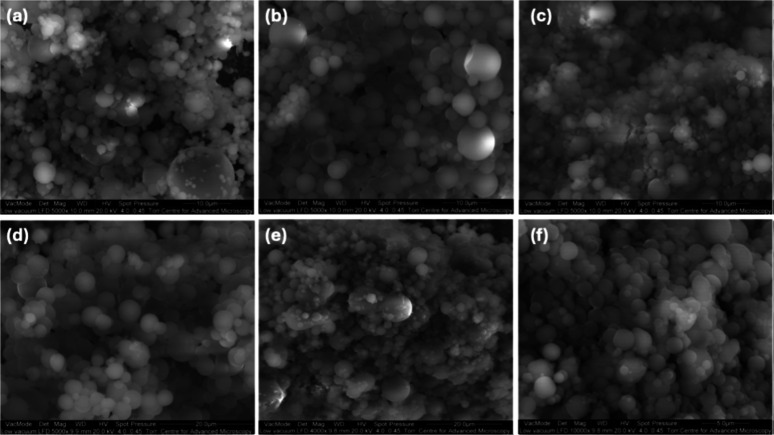
Scanning electron microscopy images of CFX/CDA 1:1 (a), 1:2 (b), 2:1 (c) and CFX/CA 1:1 (d), 1:2 (e) and 2:1 (f).

#### 3.2.2 Analysis of CFX–Bile Acid Molecular Interaction

FTIR spectroscopy was used to investigate the molecular interactions between CFX and CA within the prepared dispersions by either milling or spray drying (Figure 2). Spectra were recorded for the pure components (CFX and CA) and their mixtures at three molar ratios (1:1, 1:2, and 2:1). Key spectral regions analyzed included 1600–1800 cm^–1^ (C≡O vibrations) and 1000–1200 cm^–1^ (fingerprint region, including potential C–F and C–O vibrations). CFX is known to form a zwitterionic structure under specific conditions, with the CA (–COOH) group losing a proton and the piperazine ring gaining one. This dual nature can result in complex interactions with excipients like CA, especially in systems undergoing mechanical or thermal processing. The absence of a distinct carboxylic acid peak (∼1710 cm^–1^) in the spectrum of pure CFX confirms the zwitterionic nature of the molecule. Instead, asymmetric and symmetric vibrations of the ionized carboxylate group (COO^–^) appear at approximately 1585 cm^–1^ and 1375 cm^–1^, respectively. The peak at 1585 cm^–1^ disappeared, indicating the formation of free COOH with a potential to form hydrogen bonds. In the aromatic ketone region (∼1610 cm^–1^), pure CFX exhibits a strong C≡O stretching peak. In the mixtures, this peak shifts to 1615 cm^–1^ in milled samples and to 1618 cm^–1^ in spray-dried samples, suggesting hydrogen bonding or electronic environment changes due to interactions with CA. The region near 1700–1750 cm^–1^ displays overlapping contributions from CA’s carbonyl groups and any residual –COOH groups from CFX. Peaks near 1720 cm^–1^ in pure CA shift to 1715 cm^–1^ in spray-dried mixtures, highlighting dynamic hydrogen bonding between the components. For the C–F bond vibrations (∼1300–1100 cm^–1^), pure CFX shows sharp peaks near 1240 cm^–1^. In milled samples, these peaks broaden and shift slightly to 1235 cm^–1^. In spray-dried samples, further broadening and a shift to 1230 cm^–1^ are observed, reflecting possible interactions between fluorine atoms and the hydroxyl groups of CA.

**Figure 2:**
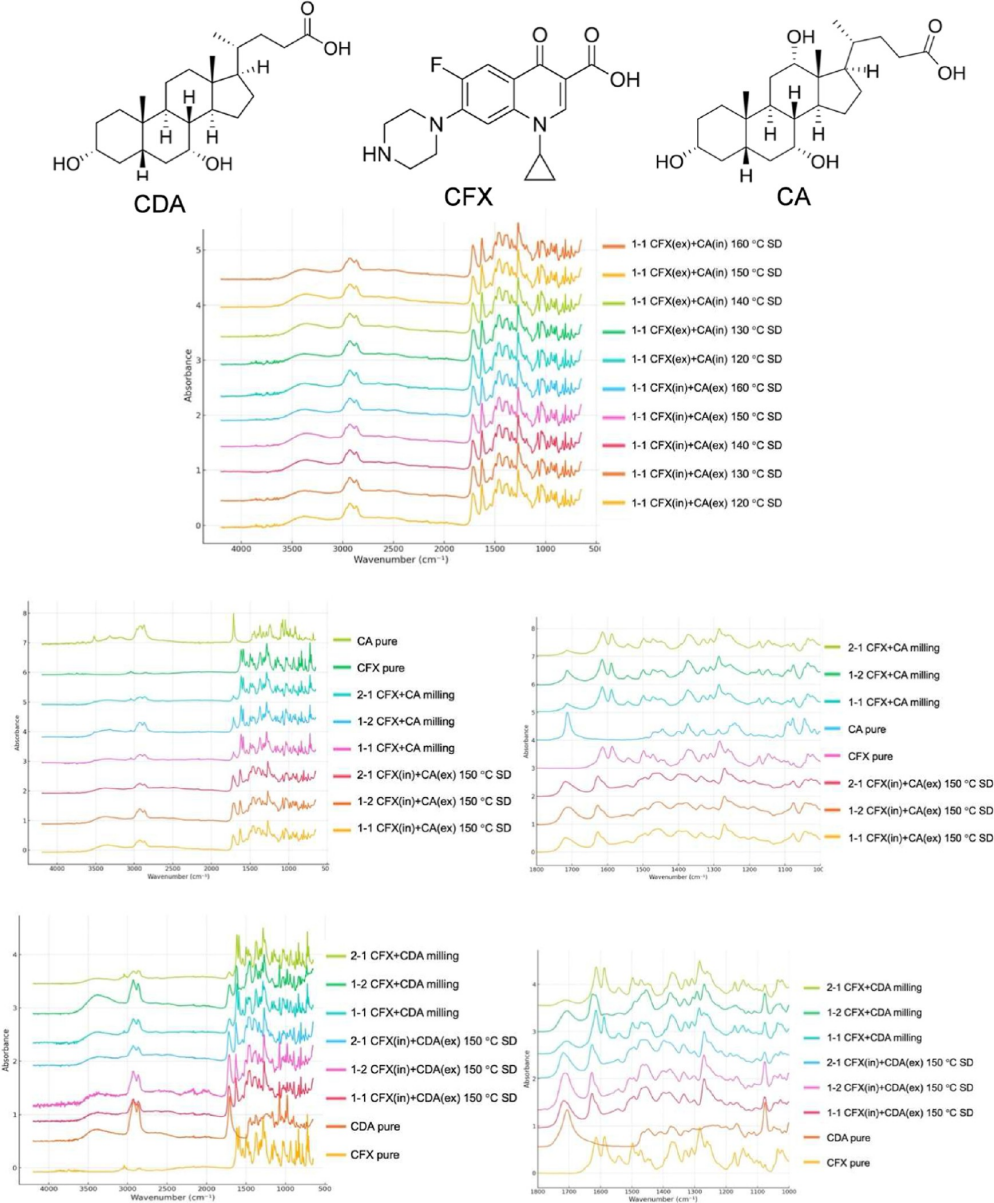
FTIR spectra of CFX/CA and CFX/CDA spray-dried and milled dispersions.

Specifically, the shift in the carbonyl (C≡O) stretching vibration from ∼1610 cm^–1^ (in pure CFX) to higher wavenumbers (1615–1618 cm^–1^) in the mixtures is consistent with the hydrogen-bond formation between CFX and the bile acids. This interaction weakens the resonance delocalization between the carbonyl group and the adjacent aromatic system, causing the C≡O bond to adopt more single-bond character and vibrate at a higher frequency.

In addition, hydrogen bonding introduces a localized electronic redistribution around the C≡O group, altering its dipole moment and reducing conjugation with neighboring groups. These effects are well-documented in solid-state FTIR analysis of hydrogen-bonded systems and support our interpretation that stronger specific interactions (particularly in CDA systems) contribute to the observed spectral changes. These findings are also consistent with the broader shift and disappearance of the COO^–^ band (∼1585 cm^–1^), indicating the modification of ionic and hydrogen-bonding interactions in the dispersion.

No significant features are present in the high wavenumber region (∼3300–4000 cm^–1^), confirming that references to the stretching of O–H in an earlier analysis were incorrect. The observed data emphasize interactions predominantly in the lower wavenumber regions associated with ionic and hydrogen-bonding mechanisms. CFX’s zwitterionic nature plays a central role in its interactions with CA. The absence of a distinct CA peak and the presence of COO^–^ vibrations confirm its betaine-like structure, where electronic effects dominate. Milling induces initial hydrogen bonding, as evidenced by minor shifts and peak broadening. In contrast, spray drying amplifies these effects, likely due to the enhanced mobility of molecules under thermal conditions, enabling stronger hydrogen bonds and ionic interactions. Examination of the spectra for samples prepared using inlet temperature values and nozzle configuration showed almost identical spectra, which confirm uniform formation and lack of differences in intermolecular interactions.

A similar pattern was observed in CDA mixtures with CFX in terms of peak shifts. A key difference was the disappearance of the peak at ∼1585 cm^–1^ in CFX when combined with CDA but not with CA. This can be attributed to structural differences between CDA and CA. CDA has hydroxyl groups at positions 3α and 7α, while CA has additional hydroxylation at position 12α. This structural variation influences the spatial arrangement and hydrogen-bonding capabilities of the molecules. The specific configuration of CDA may facilitate stronger or more specific interactions with CFX, leading to the observed spectral changes, whereas the presence of the extra hydroxyl group in CA alters its interaction pattern with CFX, resulting in the retention of the ∼1585 cm^–1^ peak. These spectral shifts suggest stronger hydrogen bonding between the carboxyl and hydroxyl groups of CDA and the fluoroquinolone ring of CFX, possibly via bidentate interactions. In contrast, CA lacks the same polar arrangement and hydroxyl density, reducing its hydrogen-bond donor/acceptor potential. These findings highlight that CDA engages in stronger molecular interactions with CFX than CA, as evidenced by the disappearance of the COO^–^ band and greater C≡O peak shift. Although CA has a solubility parameter more closely matching that of CFX, this global parameter does not account for the directionality or geometry of specific interactions. CDA, with hydroxyl groups at 3α and 7α positions, likely forms bidentate hydrogen bonds with CFX’s carboxylic and ketone groups. In contrast, CA’s additional 12α-hydroxyl may introduce steric hindrance, reducing hydrogen-bond efficiency. This suggests that spatial compatibility and interaction specificity outweigh simple thermodynamic similarity. Specifically, the 12α-hydroxyl group in CA increases its polarity and raises its Hildebrand solubility parameter, which superficially suggests better thermodynamic compatibility with CFX. However, this group also introduces steric bulk and alters the spatial configuration of the bile acid, potentially hindering optimal hydrogen bonding with CFX. This may explain why CDA, despite having a lower solubility parameter, forms more favorable directional interactions, resulting in stronger molecular cohesion and superior formulation performance.

#### 3.2.3 Thermal Analysis of Prepared Dispersions

Thermal analysis results indicated *T*_g_ values ranging from 92.79 to 127.55 °C across all formulations (Figure 3 and Table 2). Most spray-dried samples displayed multiple glass-transition events, consistent with the formation of glassy suspensions—systems containing two or more amorphous phases that are phase-separated but lack crystallinity. This interpretation is supported by the absence of sharp melting endotherms or recrystallization peaks across the tested range, ruling out residual crystallinity and confirming that the transitions are not thermal decomposition or solvent loss. The observed *T*_g_ values deviated significantly from predictions based on the Fox and Gordon–Taylor models (eqs 2 and 3)^22,23^ commonly applied to assess the thermodynamic miscibility of binary amorphous systems, suggesting nonideal mixing and partial immiscibility. In several formulations, particularly 2:1 CFX–CA and milled samples, transitions merged into a single broad *T*_g_ region, indicating a higher degree of miscibility. These observations are consistent with the amorphous behavior in some systems and amorphous suspensions in others, where physical mixing occurs without complete molecular-level miscibility.

1 $$\frac{1}{T_{g}}=\frac{w_{1}}{T_{g1}}+\frac{w_{2}}{T_{g2}}$$

where *T*_g_ is the predicted glass-transition temperature of the mixture, w_1_ and w_2_ are the weight fractions of CFX and CA or CDA, and *T*_g1_ and *T*_g2_ are the *T*_g_ values of the pure components.

2 $$T_{g}=\frac{w_{1}T_{g1}+kw_{2}T_{g2}}{w_{1}+{kw}_{2}}$$

**Figure 3:**
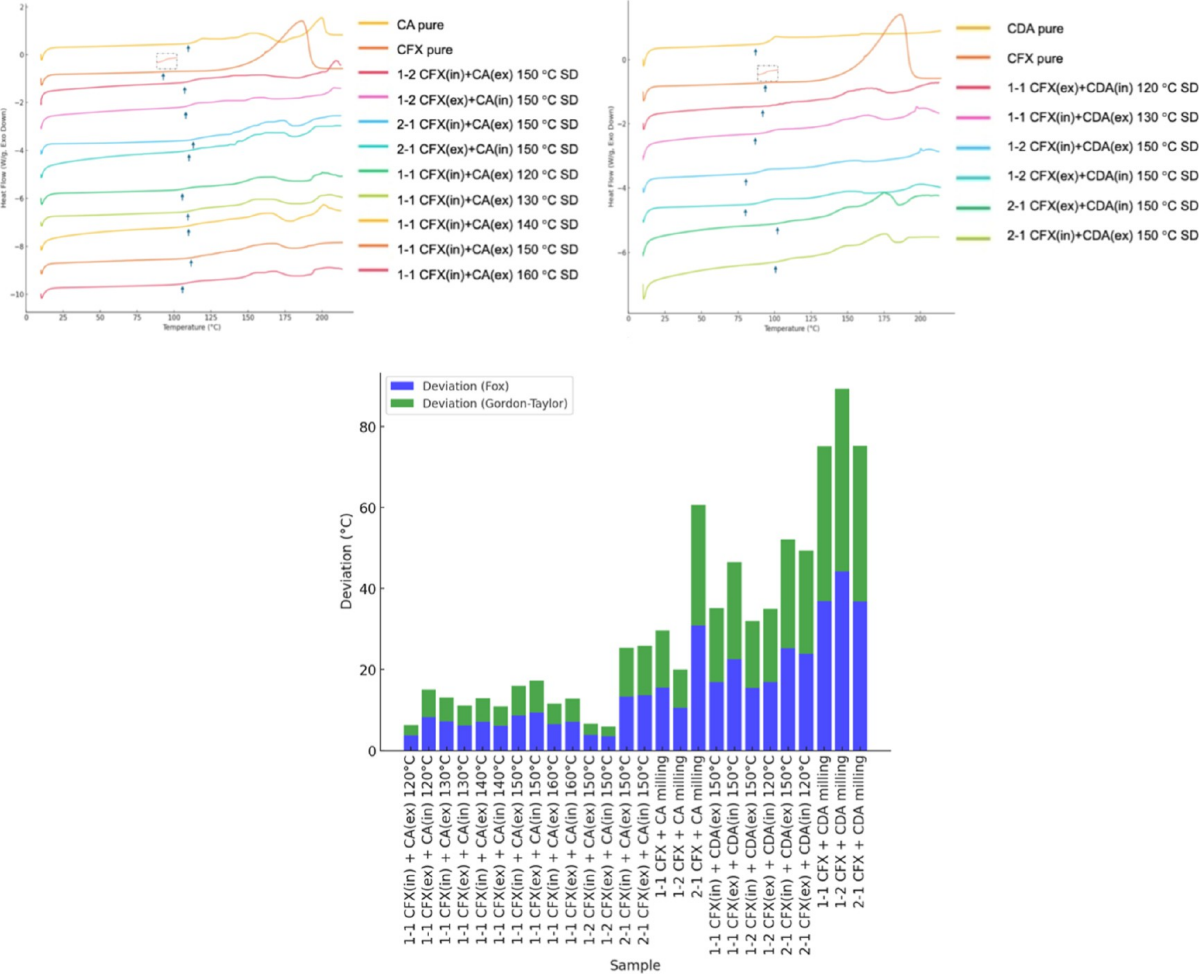
(Top) DSC thermograms showing CA and CDA samples as measured by DSC. Arrows indicate glass-transition events. Multiple *T*_g_ values are visible in several samples, consistent with glassy suspensions—i.e., phase-separated amorphous systems. The absence of melting or crystallization peaks confirms the lack of residual crystallinity. (Bottom) Deviation of observed *T*_g_ from predicted *T*_g_ values using the Fox and Gordon–Taylor equations. Larger deviations, particularly in drug-rich and milled samples, suggest nonideal mixing behavior and stronger molecular interactions than that predicted by compositional models. CDA-based formulations and milled dispersions show the greatest *T*_g_ elevation, consistent with enhanced amorphization.

**Table 2:** Summary of Thermal Analysis Data Showing Detected Thermal Transitions

Sample	yield (%)	*T*_g_ (°C) mean ± SD	predicted *T*_g_ °C (Fox)	predicted *T*_g_ °C (Gordon–Taylor)
1–1 CFX(in) + CA(ex) 120 °C	82.29	108.66 ± 0.80	104.88	106.21
1–1 CFX(ex) + CA(in) 120 °C	82.53	113.06 ± 0.15	104.88	106.21
1–1 CFX(in) + CA(ex) 130 °C	83.74	112.08 ± 0.64	104.88	106.21
1–1 CFX(ex) + CA(in) 130 °C	84.72	111.05 ± 0.23	104.88	106.21
1–1 CFX(in) + CA(ex) 140 °C	82.35	111.98 ± 0.26	104.88	106.21
1–1 CFX(ex) + CA(in) 140 °C	85.85	110.98 ± 0.46	104.88	106.21
1–1 CFX(in) + CA(ex) 150 °C	80.98	113.49 ± 0.17	104.88	106.21
1–1 CFX(ex) + CA(in) 150 °C	81.34	114.15 ± 0.10	104.88	106.21
1–1 CFX(in) + CA(ex) 160 °C	82.29	111.34 ± 1.47	104.88	106.21
1–1 CFX(ex) + CA(in) 160 °C	84.67	111.92 ± 0.19	104.88	106.21
1–2 CFX(in) + CA(ex) 150 °C	83.32	112.74 ± 0.32	108.89	110.04
1–2 CFX(ex) + CA(in) 150 °C	87.61	112.38 ± 0.10	108.89	110.04
2–1 CFX(in) + CA(ex) 150 °C	79.51	114.16 ± 0.20	100.89	102.12
2–1 CFX(ex) + CA(in) 150 °C	73.83	114.45 ± 0.13	100.89	102.12
1–1 CFX + CA milling	93.00	120.36 ± 0.69	104.88	106.21
1–2 CFX + CA milling	95.50	119.44 ± 0.22	108.89	110.04
2–1 CFX + CA milling	97.40	131.77 ± 0.76	100.89	102.12
1–1 CFX(in) + CDA(ex) 150 °C	82.09	97.10 ± 0.13	80.30	78.80
1–1 CFX(ex) + CDA(in) 150 °C	78.96	102.79 ± 0.23	80.30	78.80
1–2 CFX(in) + CDA(ex) 150 °C	76.42	92.57 ± 0.43	77.17	76.06
1–2 CFX(ex) + CDA(in) 120 °C	77.35	94.04 ± 0.15	77.17	76.06
2–1 CFX(in) + CDA(ex) 150 °C	82.95	109.13 ± 0.36	83.90	82.28
2–1 CFX(ex) + CDA(in) 120 °C	71.32	107.75 ± 0.13	83.90	82.28
1–1 CFX + CDA milling	96.50	117.12 ± 0.31	80.30	78.80
1–2 CFX + CDA milling	97.70	121.28 ± 0.23	77.17	76.06
2–1 CFX + CDA milling	95.20	120.67 ± 0.22	83.90	82.28

where *k* is the interaction parameter defined as

3 $$k=\frac{\rho_{1}T_{g1}}{\rho_{2}T_{g2}}$$

where ρ1 and ρ2 are the densities of the pure components.

Analysis of spray-dried samples showed that *T*_g_ for pure CFX is 93 °C, CA 117 °C, and CDA 72 °C. The density values were adapted from the Royal Society of Chemistry ChemSpider. The observed *T*_g_ values were consistently higher than those predicted by both models, indicating stronger intermolecular interactions than those accounted for by simple additive mixing rules. The Gordon–Taylor model, which incorporates density differences, provided slightly better alignment with experimental data compared to the Fox equation. The influence of bile acid type and CFX/bile acid ratio on *T*_g_ was analyzed to assess miscibility trends. 1–1 CFX + CA exhibited observed *T*_g_ values ranging from 108.66 to 114.15 °C, which were higher than the predicted values (104.88 °C from Fox and 106.21 °C from Gordon–Taylor). 2–1 CFX + CA displayed even higher *T*_g_ values (114.16 °C–131.77 °C) compared to predicted values (∼100.89 °C–102.12 °C). This trend was most pronounced in the milled samples. In contrast, 1–1 CFX + CDA had lower *T*_g_ values (97.10 °C–102.79 °C) than CFX + CA. The predicted *T*_g_ values were lower (∼80.3 °C from Fox and ∼78.8 °C from Gordon–Taylor), suggesting some additional stabilizing interactions. 2–1 CFX + CDA formulations exhibited higher *T*_g_ values (109.13 °C–120.67 °C) than 1–1, showing that higher CFX content stabilizes the amorphous phase. Spray-drying inlet temperature (120 °C–160 °C) had minimal impact on *T*_g_ across all formulations. However, milled samples consistently exhibited the highest *T*_g_ values across all compositions. For CA formulations, milling resulted in *T*_g_ increases of up to 15 °C compared to spray-dried samples, whereas for CDA formulations, milling resulted in an even greater increase, with the observed *T*_g_ values nearing 120 °C despite predictions around 80 °C. The observed deviations suggest nonideal mixing behavior, possibly due to additional molecular interactions beyond simple component blending. CDA formulations exhibited greater deviations from predicted values (up to +10 °C), suggesting intermolecular interactions that were stronger than expected. CA formulations had smaller deviations, which may suggest that CA interacts less strongly with CFX compared to CDA. The deviation arises due to nonideal mixing between CFX and the bile acids (CA and CDA), where the assumption of uniform, random molecular dispersion is not fully met. The presence of strong specific interactions, such as directional hydrogen bonding, disrupts the additive behavior expected in ideal mixtures. In systems where phase separation or partial miscibility occurs—such as physical dispersions or glassy suspensions—the observed *T*_g_ can diverge significantly from predicted values. Moreover, preferential hydrogen bonding between CDA and CFX, as indicated by FTIR and SHAP analyses, likely restricts molecular mobility, leading to a higher observed *T*_g_ than predicted. In contrast, in partially amorphous or phase-separated systems, such as those with CA at low drug ratios, this effect is less pronounced.

Higher CFX content (2–1 ratio) led to increased *T*_g_, suggesting greater molecular rigidity and amorphous phase stabilizations. CFX + CA formulations exhibited a higher *T*_g_ than CFX + CDA dispersions. Milled samples displayed the highest *T*_g_ values, showing the role of processing in enhancing molecular interactions. The Fox and Gordon–Taylor equations underestimated *T*_g_ values, suggesting additional molecular interactions beyond simple compositional effects. Together with the XRPD data (Section 3.2.4), which demonstrated broad amorphous halos in most formulations with minor residual crystalline peaks in some compositions, these results indicate that the systems ranged from fully amorphous to partially amorphous dispersions. Formulations showing a single glass-transition temperature (*T*_g_) and absence of crystalline reflections may be classified as co-amorphous systems, consistent with molecular-level miscibility. In contrast, systems with residual crystallinity are best described as partially amorphous binary dispersions, where the degree of amorphization is influenced by drug loading and the processing method.

#### 3.2.4 Analysis of Crystallinity Using XRPD

XRPD patterns of the binary spray-dried formulations (Figure 4) reveal broad diffuse halos spanning 5–50° 2θ, characteristic of amorphous materials. Compared to the sharp, well-defined peaks observed in the crystalline reference samples (CFX, CA, and CDA), the coformulated dispersions exhibited a significant reduction in crystallinity. In most formulations, especially at 2:1 and milled ratios, crystalline peaks were absent or reduced to near-baseline intensity, suggesting a predominantly amorphous structure. A residual diffraction peak near 25.18° 2θ, characteristic of crystalline CFX, was occasionally observed in some 1:1 and 1:2 CFX–CA formulations but at significantly reduced intensity. This peak diminished further in 2:1 samples, indicating that higher CFX content enhanced amorphization, possibly by promoting better dispersion within the bile acid matrix. The crystalline peaks of CA and CDA were not evident in any mixture, indicating their complete amorphization or dissolution during spray drying. Together, the XRPD data suggest that the systems formed were largely amorphous with minor residual crystallinity in some lower drug-loading formulations. These findings are consistent with the thermal analysis data, supporting the formation of predominantly amorphous systems or, in some cases, glassy suspensions, where phase-separated amorphous domains may persist due to incomplete molecular-level miscibility.

**Figure 4:**
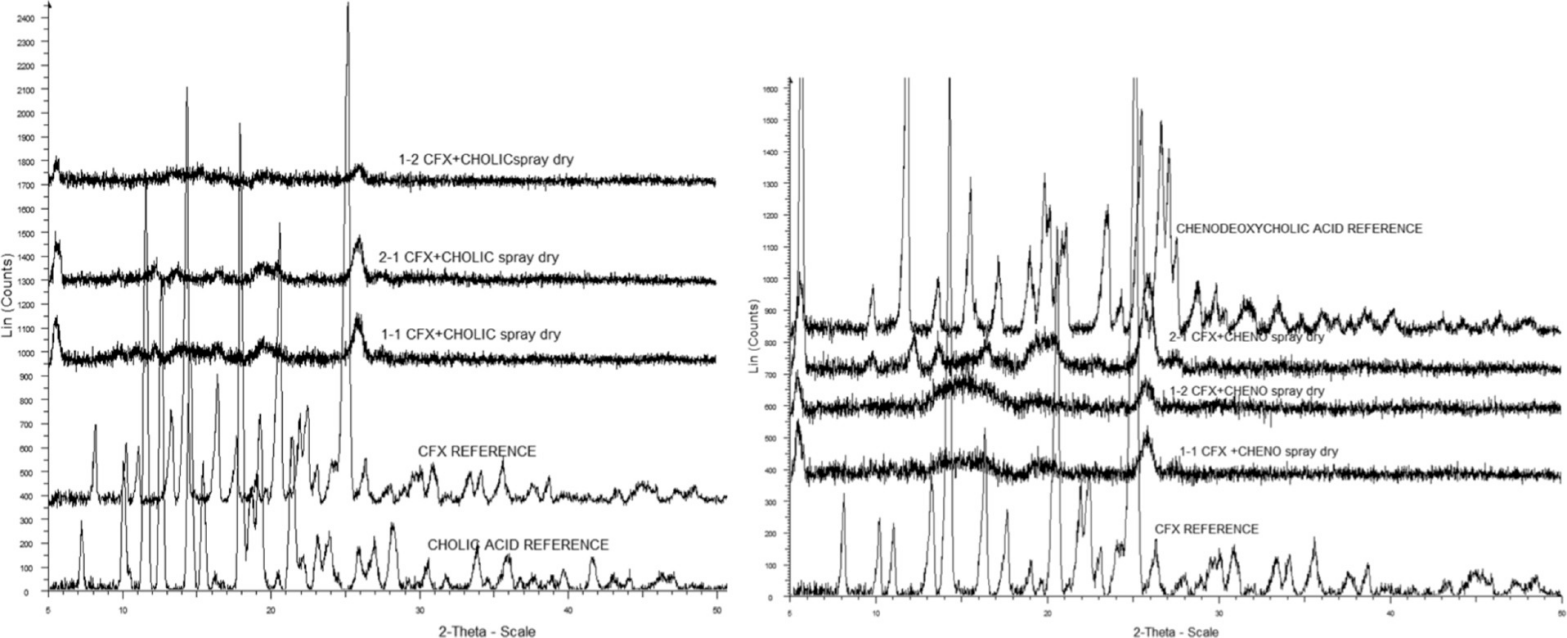
XRPD scans showing CFX–CA and CFX–CDA dispersions.

#### 3.2.5 Dissolution and Solubility Measurement of Prepared Dispersions

CFX is considered to be poorly soluble in water; its aqueous solubility is equal to 0.09 mg/mL at 37 °C.^24^ CA is also insoluble in water (solubility is 0.175 mg/mL^25^), while CDA solubility is 0.0899 mg/mL.^26^ Figure 5 illustrates the saturation solubility profiles of spray-dried CFX with CA. The graphs show selected formulations; for full measurements, refer to Supporting Information. It can be seen that in CA formulations there was a gradual increase in the concentration reflected by the higher concentration of CFX from 1 to 24 h. The opposite was seen in CDA in which there was a drop in the concertation after 24 h. This trend was consistent across all ratios for spray-dried samples. Interestingly, this trend was not seen in milled samples, as the samples showed similar values after 1 and 24 h, suggesting that the amounts achieved were close to saturation. There was a clear enhancement of the spray-dried dispersions compared to milled samples. Compared to pure CFX, the spray-dried dispersions exhibited a significant increase in solubility, ranging from 0.3 to 0.5 mg/mL, approximately 4-fold higher than the crystalline reference CFX, which showed a saturation solubility below 0.1 mg/mL. The best solubility was achieved at 0.5 mg/mL, attributed to the higher-energy amorphous state of the spray-dried formulations, which facilitates rapid dissolution due to increased Gibbs free energy. When averaging across all formulations, the solubility of CFX seemed higher when CA was used as the coformer. The drop in the solubility of CFX from CFX–CDA dispersions after 24 h may suggest possible recrystallization.

**Figure 5:**
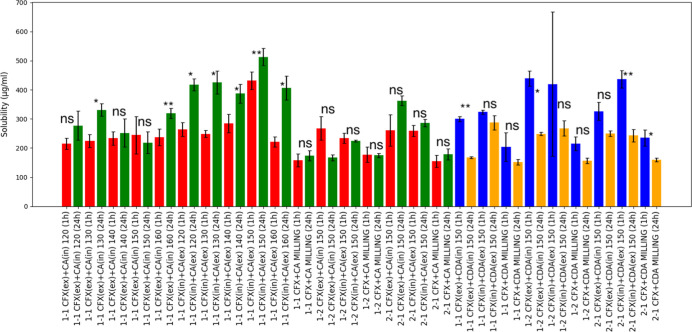
Dissolution and solubility measurements of CFX–CA and CFX–CDA dispersions measured at 1 and 24 h at pH 6.8 (phosphate buffer). (*) refers to *p* < 0.05, (**) refers to *p* < 0.01, and ns = no significant difference. Pairs were compared using paired *t* test analysis of 1 h vs 24 h solubility of the same sample. The color code for the bars was varied to facilitate the identification of CDA vs CA samples.

To investigate the influence of spray-drying nozzle configuration on solubility outcomes, formulations with identical composition, molar ratio, and inlet temperature were compared (Figure 6). Matched pairs prepared using internal and external nozzle configurations were statistically evaluated by paired *t* test. To ensure accuracy, inlet temperature was included in the grouping, avoiding the confounding effect of temperature variation. The results revealed that in several formulations, particularly those containing CA, internal nozzle configurations consistently resulted in higher solubility compared to their external counterparts. Notably, different combinations showed statistically significant differences (*p* < 0.05), with the internal nozzle of CFX enhancing the solubility values by 20–40% relative to the external feed. This suggests improved molecular dispersion or droplet drying dynamics when CFX is introduced via the internal nozzle, potentially enhancing miscibility during solvent evaporation. These findings highlight the role of nozzle configuration as a critical process variable in optimizing spray-dried dispersions.

**Figure 6:**
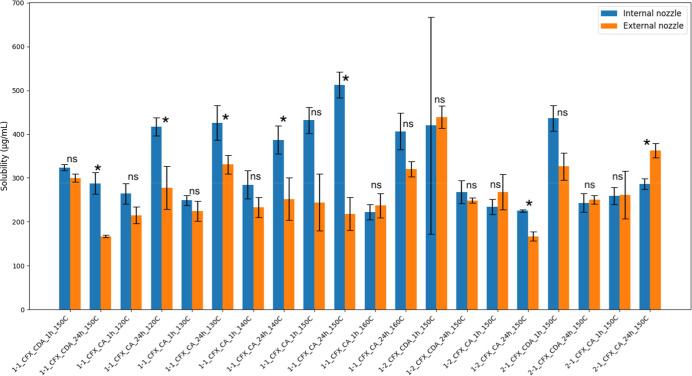
Solubility of co-spray-dried CFX–bile acid formulations prepared using internal or external nozzle configurations at various inlet temperatures. Matched pairs (internal vs external) were spray-dried using a three-fluid nozzle, where either CFX or the bile acid component CA or CDA was fed through the internal or external nozzle, respectively. Each group represents a fixed molar ratio (1:1, 1:2, or 2:1), bile acid type, time point (1 or 24 h), and inlet temperature (120–160 °C). Bars represent the mean solubility (μg/mL) ± SD (*n* = 3). Statistical significance between internal and external nozzle conditions was assessed by paired *t* test, * = significant difference at *p* < 0.05, ns = not significant.

#### 3.2.6 In Vitro Lung Deposition Using Andersen Cascade Impactor (ACI)

The aerodynamic performance of the formulations was assessed by evaluating key parameters, as summarized in Figure 7 and Table 3. Statistical analysis (Tukey HSD) revealed significant differences across the formulations, highlighting the role of bile acid type and component ratios on the aerosolization properties. As can be seen, the CDA-based formulations showed improved aerosolization compared to the CA-based dispersions. 1–1 CFX–CDA demonstrated a lower CPF (69.19 ± 0.23%) compared to 1–1 CFX–CA (73.07 ± 1.13%), which was statistically significant (*p* < 0.05). Similarly, FPF was higher for 1–1 CFX–CDA (30.81 ± 0.23%) than for 1–1 CFX–CA (26.93 ± 1.13%).

**Figure 7:**
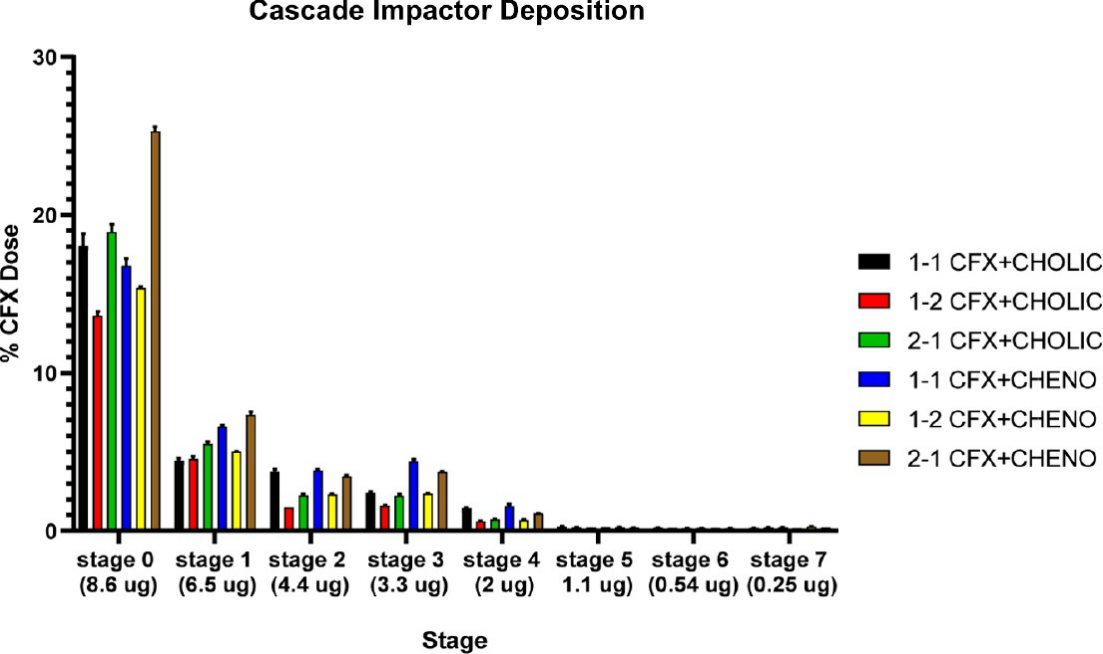
Andersen cascade impactor showing deposition data of spray-dried dispersions of CFX with CA and CDA.

**Table 3:** Lung Deposition by Andersen Cascade Impactor (ACI); All Datasets Per Each Test Are Statistically Different (*p* < 0.05)

Sample	CPF (%)	ED (mg)	EFPF (%)	FPD (mg)	FPF (%)	GSD	MMAD (μm)
1–1 CFX + CDA	69.19 ± 0.23	3.60 ± 0.05	0.01 ± 0.04	1.11 ± 0.01	30.81 ± 0.23	1.72 ± 0.01	5.89 ± 0.04
1–1 CFX + CA	73.07 ± 1.13	3.12 ± 0.12	0.01 ± 0.06	0.84 ± 0.02	26.93 ± 1.13	1.74 ± 0.01	6.19 ± 0.08
1–2 CFX + CDA	77.00 ± 0.11	2.64 ± 0.02	0.02 ± 0.03	0.61 ± 0.00	23.00 ± 0.11	1.76 ± 0.00	6.28 ± 0.01
1–2 CFX + CA	80.69 ± 0.25	2.16 ± 0.01	0.02 ± 0.04	0.42 ± 0.01	19.31 ± 0.25	1.76 ± 0.01	6.43 ± 0.03
2–1 CFX + CDA	78.60 ± 0.30	4.93 ± 0.06	0.01 ± 0.01	1.06 ± 0.03	21.40 ± 0.30	1.64 ± 0.00	6.53 ± 0.01
2–1 CFX + CA	80.68 ± 0.31	3.11 ± 0.03	0.01 ± 0.05	0.60 ± 0.01	19.32 ± 0.31	1.68 ± 0.01	6.58 ± 0.02

For the 1–2 formulations, a notable increase in CPF was observed for both bile acids (77.00 ± 0.11% for CDA and 80.69 ± 0.25% for CA), with significant differences between the two (*p* < 0.05). The reduced FPF values (23.00 ± 0.11% for 1–2 CFX–CDA and 19.31 ± 0.25% for 1–2 CFX–CA) reflect a higher proportion of coarse particles, likely due to the increased ratio of CA in the formulation. The 2–1 formulations showed the highest CPF values, reaching 78.60 ± 0.30% for 2–1 CFX–CA and 80.68 ± 0.31% for 2–1 CFX + CA, which were significantly different (*p* < 0.05). However, the ED was higher for 2–1 CFX–CDA (4.93 ± 0.06 mg) compared to 2–1 CFX + CA (3.11 ± 0.03 mg), indicating more effective delivery of the active drug when using CDA as a bile acid. Statistically significant differences (*p* < 0.05) were also observed in MMAD values across the formulations. The 1–1 CFX–CDA formulation exhibited a smaller MMAD (5.89 ± 0.04 μm) compared to 1–1 CFX–CA (6.19 ± 0.08 μm), reflecting improved particle size distribution with CDA. Similar trends were observed for the 1–2 and 2–1 formulations, where MMAD values for CDA-based formulations were consistently smaller than those with CA.

#### 3.2.7 Determination of MIC

The MIC against the clinical isolates of *Klebsiella pneumonia* for all formulations was determined using the broth microdilution technique following CLSI and EUCAST guidelines. With the exception of CDA (reference material), all other formulations exhibited strong minimal inhibitory antimicrobial activities (Figure 8). All antimicrobial assays were conducted using serial dilutions based on the CFX content in each formulation, ensuring a direct comparison across CFX, CFX–CA, and CFX–CDA groups. As can be seen, MIC assays confirmed that all formulations retained strong antibacterial activity against *Klebsiella pneumonia*. While pure CFX exhibited the lowest OD600 values, the CFX–CDA formulation showed comparable inhibition, particularly at lower concentrations, suggesting that CDA maintained the antimicrobial efficacy of CFX. In contrast, CFX–CA showed slightly higher OD600 values, indicating a less favorable interaction. Importantly, both CFX–bile acid formulations offered added advantages in solubility, aerosolization, and potential stability, with CDA emerging as the more effective coformer. These findings support the viability of bile acid-based systems as inhalable alternatives that preserve antimicrobial potency while improving the physicochemical performance.

**Figure 8:**
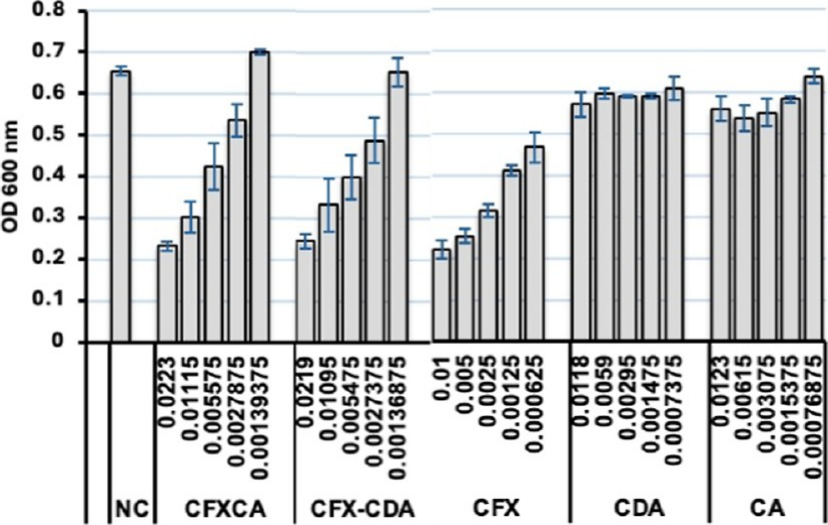
MIC of formulations against clinically isolated *Klebsiella pneumonia*. NC, negative control, CFX–CA, CFX–CDA, CFX, CDA, and CA.

### 3.3 In Vivo Toxicological Results

A daily visual assessment of the rats throughout the course of the experiment revealed that none of the rats was showing any signs of toxicity, including behavioral change, laying down, being isolated, eye discharges, profound salivation, mortality, piloerection, cannibalism, diarrhea, or anorexia. The body weight, which was measured at day 1 (first day of the dosing), day 8, and at the end of the treatment (day 15), revealed a steady increase throughout the course of the treatments (Table 4). All groups gained weight during the study period. Weight trajectories for CFX–CA and CFX–CDA groups were broadly similar to that of CFX-L/H, with no statistically significant differences attributable to bile acid coformers.

**Table 4:** Body Weight (g) of the Treated Rats at Different Time Intervals, Including Day 1, Day 8, and Day 15 of the Experiment^a^

	time		
treatment	day 1	day 8	day 15
NC	216.67 ± 14.43^A^	245.33 ± 31.02^AB^	291.17 ± 7.32^A^
CFX-L	248.67 ± 35.79^A^	258.67 ± 33.48^AB^	291.50 ± 41.5^A^
CFX-H	223.00 ± 43.55^A^	283.67 ± 46.91^A^	272.67 ± 17.55^A^
CFX–CAL	265.00 ± 91.78^A^	261.00 ± 33.86^AB^	281.00 ± 28.68^A^
CFX–CAH	228.33 ± 63.31^A^	259.33 ± 32.62^AB^	286.00 ± 43.27^A^
CFX–CDAL	210.00 ± 5.00^A^	217.67 ± 7.37^B^	236.33 ± 4.50^A^
CFX–CDAH	205.33 ± 10.50^A^	240.00 ± 21.79^AB^	248.33 ± 50.01^A^

a NC, negative control, CFX-L (ciprofloxacin-low dose), CFX-H (ciprofloxacin-high dose), CFX–CAL (CFX–CA-low dose), CFX–CAH (CFX–CA-high dose), CFX–CDAL (CFX–CDA-low dose), CFX–CDAH (CFX–CDA-high dose). Superscripts on the right of the means indicate statistical differences (*P* < 0.05) between the groups. Values are means ± standard deviations for *N* = 3. Statistical analysis was done using one-way ANOVA.

After being sacrificed (at day 15), the organs, including the spleen, kidneys, testicles, liver, and the heart, were collected aseptically and carefully checked for any visible changes in the shape and texture of the organs; there were not any observed changes in the structure of the organs after being checked; then, the weight of the organs was measured, and the results showed that the weight of kidneys was significantly lower in the CFX–CDAH-treated animals. However, the weight of the heart was also lower in the CFX-L, CFX-H, CFX–CDAL, and CFX–CDAH groups when compared to the control negative group (Table 5). Spleen and liver weights showed no significant differences among any groups. In contrast, kidney and testicle weights were significantly lower in CFX–CDAL compared with CFX-L, although this reduction was not observed in CFX–CDAH. For the heart, both CFX–CDAL and CFX–CDAH were significantly lower than CFX-L/H, while CA formulations did not differ significantly from the drug controls. These findings suggest a possible dose-related effect of CDA on renal and cardiac mass that warrants further investigation, although histopathology revealed no structural abnormalities.

**Table 5:** Organ Weights (g) of the Rat at the End of the Experiment^a^

	Organs				
treatments	spleen	kidneys	testicles	liver	heart
NC	1.21 ± 0.30^A^	2.24 ± 0.20^AB^	6.57 ± 0.73^AB^	11.32 ± 1.02^A^	1.51 ± 0.07^C^
CFX-L	0.88 ± 0.33^A^	2.55 ± 0.08^A^	7.32 ± 1.88^A^	11.30 ± 2.01^A^	1.25 ± 0.10^B^
CFX-H	1.19 ± 0.27^A^	2.23 ± 0.32^AB^	7.13 ± 1.34^AB^	11.10 ± 1.61^A^	1.25 ± 0.17^B^
CFX–CAL	0.96 ± 0.19^A^	2.26 ± 0.10^AB^	6.64 ± 1.00^AB^	10.85 ± 1.84^A^	1.39 ± 0.03^BC^
CFX–CAH	1.22 ± 0.27^A^	2.30 ± 0.31^AB^	7.17 ± 0.57^AB^	11.19 ± 2.84^A^	1.31 ± 0.12^BC^
CFX–CDAL	0.86 ± 0.12^A^	1.74 ± 0.15^C^	5.4 ± 0.57^B^	8.56 ± 0.86^A^	0.99 ± 0.17^A^
CFX–CDAH	1.14 ± 0.41^A^	2.02 ± 0.15^BC^	6.38 ± 1.13^AB^	9.87 ± 1.35^A^	1.03 ± 0.09^A^

a NC, negative control, CFX-L (ciprofloxacin-low dose), CFX-H (ciprofloxacin-high dose), CFX–CAL (CFX–CA-low dose), CFX–CAH (CFX–CA-high dose), CFX–CDAL (CFX–CDA-low dose), CFX–CDAH (CFX–CDA-high dose). Superscripts on the right of the means indicate statistical differences (*P* < 0.05) between the groups. Values are means ± standard deviations for *N* = 3. Statistical analysis was done by one-way ANOVA. Superscript letters indicate results of post hoc multiple comparisons (*p* < 0.05). Groups with the same letter are not significantly different. Groups with no letters in common (e.g., A vs C) differ significantly. Mixed labels (e.g., AB, BC) indicate overlap, meaning that the group is not significantly different from either of the letter groups it shares, but the two letter groups themselves (e.g., A vs C) may still differ.

The serum biochemical tests revealed that the levels of creatinine in the CFX-H-treated group, CRP in all groups, and AST in CFX–CDAL were significantly increased when compared to the control groups. Meanwhile, the level of total bilirubin was significantly lower in all treated animals in comparison to the control negative group (Table 6). Relative to CFX-L/H, creatinine was elevated only in CFX-H but not in CA or CDA formulations. AST was markedly elevated in CFX–CDAL, and ALP was increased in CFX–CDAH; however, ALT remained stable across all of the groups. CRP was elevated in all drug-containing groups compared with NC, with the highest levels seen in CFX–CAH, but CDA groups were not higher than CFX-H. Collectively, these data indicate that CDA formulations produce mild, dose-dependent changes in hepatic enzymes, while renal function markers remain comparable to CFX.

**Table 6:** Serum Biochemical Tests of the Treated Rats with Different Concentrations of CFX–CA and CFX–CDA^a^

	tests									
treatments	urea (mmol/L)	creatinine (μmol/L)	total protein (TP) (g/L)	albumin (ALB) (g/L)	alanine aminotransferase (ALT) (U/L)	aspartate aminotransferase (AST) (U/L)	total bilirubin (BILT3) (mg/dL)	C-reactive protien (CRP4) (mg/mL)	alkaline phosphatase (ALP) (U/L)	globulin (GLB) (g/L)
NC	27.2 ± 1.9^AB^	0.23 ± 0.02^A^	6.12 ± 0.07^A^	3.78 ± 0.04^AB^	58.75 ± 0.15^A^	175.75 ± 14.1^A^	0.20 ± 0.09B	0.095 ± 0.03^A^	338.5 ± 29.5^AB^	2.0 ± 0.0^A^
CFX-L	29.4 ± 3.15^AB^	0.29 ± 0.04^AB^	6.67 ± 0.18^A^	4.72 ± 1.27B	52.6 ± 2.28^A^	176.67 ± 34.3^A^	0.08 ± 0.03^A^	0.21 ± 0.06^B^	238.3 ± 63.31^A^	2.67 ± 0.58^A^
CFX-H	31.3 ± 7.72^B^	0.33 ± 0.04^B^	6.78 ± 0.19^A^	4.21 ± 0.12^AB^	73.7 ± 17.11^A^	197.53 ± 45.0^A^	0.07 ± 0.02^A^	0.19 ± 0.05^B^	247.3 ± 38.37^A^	2.6 ± 0.58^A^
CFX–CAL	29.6 ± 0.76^AB^	0.26 ± 0.06^AB^	6.08 ± 0.02^A^	3.75 ± 0.16^AB^	86.8 ± 29.17^A^	258.57 ± 76.8^A^	0.04 ± 0.03^A^	0.26 ± 0.03^BC^	250.0 ± 119.52^A^	2.33 ± 0.58^A^
CFX–CAH	27.1 ± 2.83^AB^	0.28 ± 0.04^AB^	6.40 ± 0.29^A^	3.94 ± 0.3^AB^	77.5 ± 7.27^A^	207.07 ± 39.4^A^	0.09 ± 0.04^A^	0.3 ± 0.07^C^	261.3 ± 30.29^A^	2.0 ± 0.0^A^
CFX–CDAL	23.3 ± 0.95^A^	0.25 ± 0.03^A^	6.19 ± 0.92^A^	3.55 ± 0.55^A^	75.9 ± 33.68^A^	568.3 ± 172.8^B^	0.06 ± 0.03^A^	0.19 ± 0.01^B^	225.0 ± 22.54^A^	2.67 ± 1.15^A^
CFX–CDAH	27.0 ± 4.65^AB^	0.22 ± 0.03^A^	6.06 ± 0.04^A^	3.83 ± 0.25^AB^	64.37 ± 3.42^A^	187.2 ± 27.76^A^	0.07 ± 0.04^A^	0.18 ± 0.05^B^	398.3 ± 50.34^B^	2.33 ± 0.58^A^

a NC, negative control, CFX-L (ciprofloxacin-low dose), CFX-H (ciprofloxacin-high dose), CFX–CAL (CFX–CA-low dose), CFX–CAH (CFX–CA-high dose), CFX–CDAL (CFX–CDA-low dose), CFX–CDAH (CFX–CDA-high dose). Values are means ± standard deviations for *N* = 3. Statistical analysis was by one-way ANOVA. Superscript letters indicate the results of post hoc multiple comparisons (*p* < 0.05). Groups with the same letter are not significantly different. Groups with no letters in common (e.g., A vs C) differ significantly. Mixed labels (e.g., AB and BC) indicate overlap, meaning that the group is not significantly different from either of the letter groups it shares, but the two letter groups themselves (e.g., A vs C) may still differ.

The lipid profile test revealed that the levels of cholesterol and LDL in CFX-H, CFX–CDAL, and CDAH, and HDL in CFX–CDAL, were significantly reduced in comparison to the control negative group. CFX–CDAL reduced the total cholesterol and LDL but also lowered HDL, an unfavorable shift not seen in other groups. Most CA and CDA groups were otherwise comparable to CFX-L/H. Other treatments were found to have no substantial influences on other lipid profile tests in comparison to the negative control group (Table S1). The results showed that the RBC counts and HCT were significantly higher in most treatment groups compared with NC but were comparable across CFX-L/H and CA/CDA formulations. At a high CDA dose (CFX–CDAH), RDW measures were elevated, suggesting increased variability in the red cell size. At a low CDA dose (CFX–CDAL), total WBC counts increased with relative lymphocytosis and monocytosis compared with CFX-L/H, while CA groups showed eosinophil elevations without broader leukocyte shifts (Table S2). Regarding WBCs, there were a significant increase in the total number of WBCs, percentage of monocytes, and a significant reduction in the percentage of neutrophils in the CFX–CDAL-treated group in comparison to the negative control group. Meanwhile, the percentage of eosinophils was increased in both the CFX–CAL- and CFX–CAH-treated groups in comparison to the control negative group (Table S3).

Furthermore, histopathological observations revealed that none of the used agents had obvious toxicological effects on the renal, spleen, and skeletal muscles. Meanwhile, some minor hepatic lesions, such as mild hepatic intracytoplasmic vacuoles, mild-to-moderate lymphocytic infiltration, with central vein congestion, and prominent nucleoli had been observed. Also, there were no membrane leakage, necrosis, and/or apoptosis. Similarly, the overall structure of the liver, hepatocytes, sinusoids, portal triads, and central veins was normal. At the same time, the kidney sections of treated rats exhibited normal structures and appearances of glomeruli/tubules. The proximal convoluted tubules, distal convoluted tubules, and macula densa were intact with no Bowman’s capsule thickening and interstitial inflammation. The spleen was found to have clear normal central arteries, which are located within the lymphatic nodules, normal trabeculae, and well-defined red and white bulbs. The skeletal muscles were found to have a normal and well-defined myocyte with peripherally located nuclei (Figures 9–12). Overall, no overt toxicity was detected in the kidneys, spleen, or skeletal muscle. Liver sections across groups showed only mild, nonspecific changes (e.g., vacuolation and lymphocytic infiltration) without necrosis or apoptosis, consistent with the biochemical alterations noted above. The data demonstrated that CDA formulations produced mild, dose-dependent hepatic effects, with AST elevations at low doses and ALP increases at high doses. Importantly, no histopathological liver damage was detected, and CA-based formulations showed a safety profile broadly comparable to CFX. Nevertheless, these hepatic signals in CDA formulations highlight a translational limitation and emphasize the need for careful monitoring in longer-term inhalation studies.

**Figure 9:**
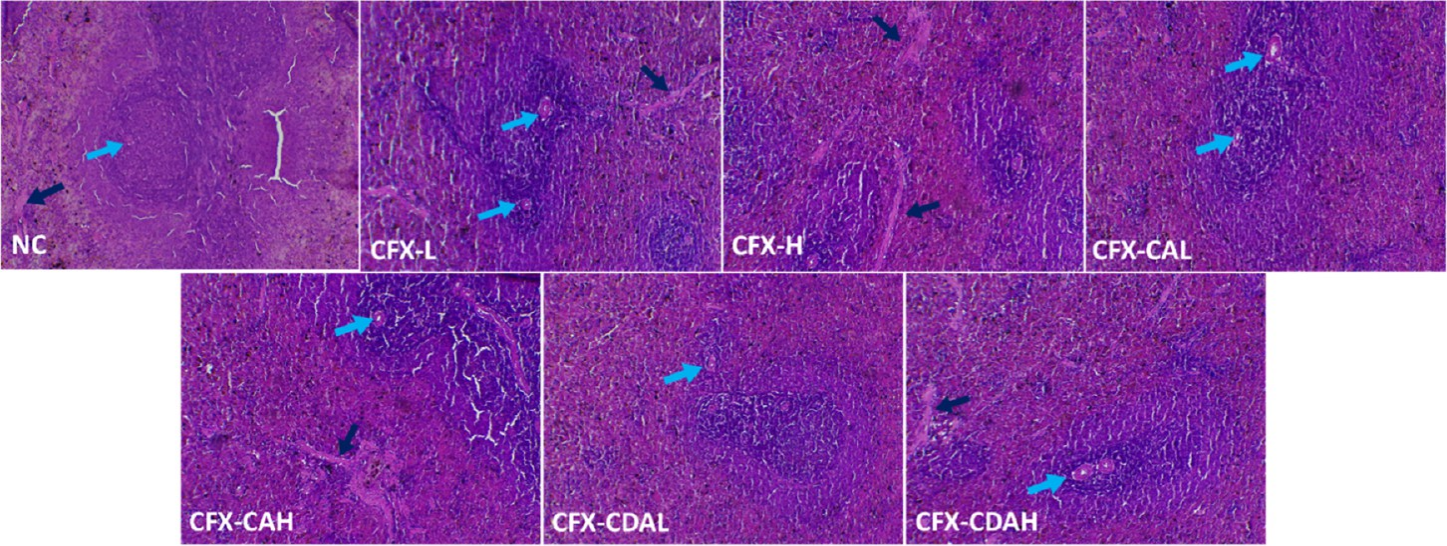
Histopathological section of the spleen of rats, which had been treated with different concentrations of CFX–CA and CFX–CDA. NC, negative control, CFX-L (ciprofloxacin-low dose), CFX-H (ciprofloxacin-high dose), CFX–CAL (CFX–CA-low dose), CFX–CAH (CFX–CA-high dose), CFX–CDAL (CFX–CDA-low dose), CFX–CDAH (CFX–CDA-high dose), CFX–CDAL shows central arteries (blue arrow), which are located within the lymphatic nodules, and trabeculae (black arrow), which are made of connective tissue.

**10:**
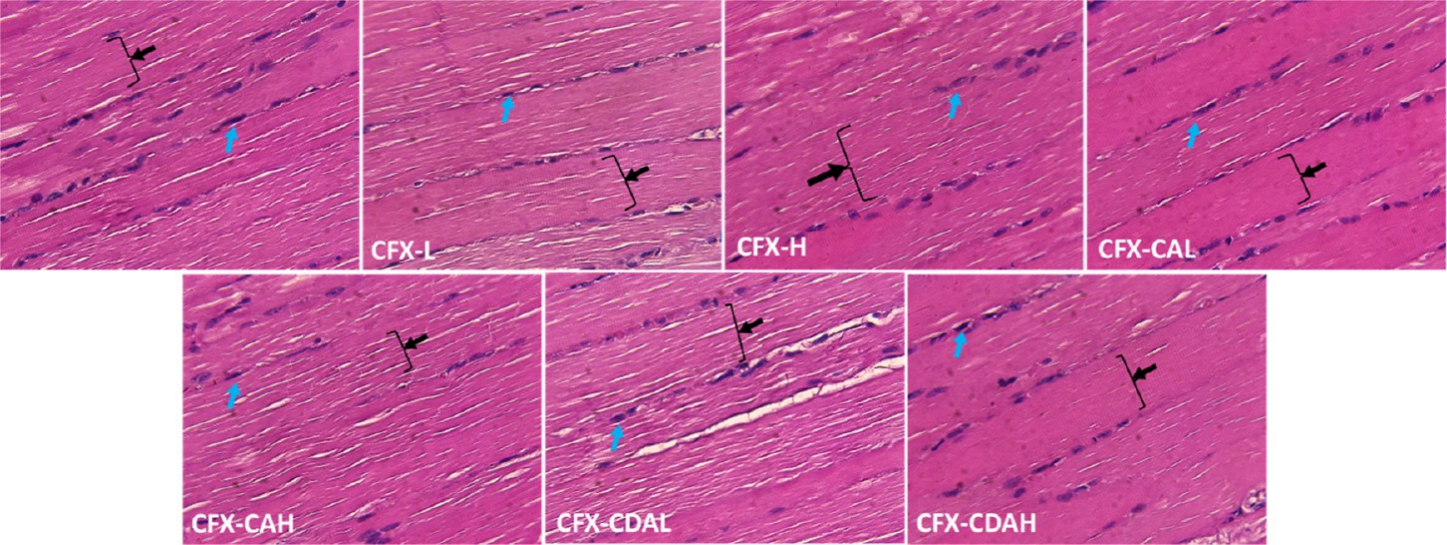
Histopathological section of the skeletal muscle of rat, which had been treated with different concentrations of CFX–CA and CFX–CDA. NC, negative control, CFX-L (ciprofloxacin-low dose), CFX-H (ciprofloxacin-high dose), CFX–CAL (CFX–CA-low dose), CFX–CAH (CFX–CA-high dose), CFX–CDAL (CFX–CDA-low dose), CFX–CDAH (CFX–CDA-high dose), CFX–CDAL shows muscle cell nucleus (blue arrow), and muscle fiber (black arrow and brace).

**11:**
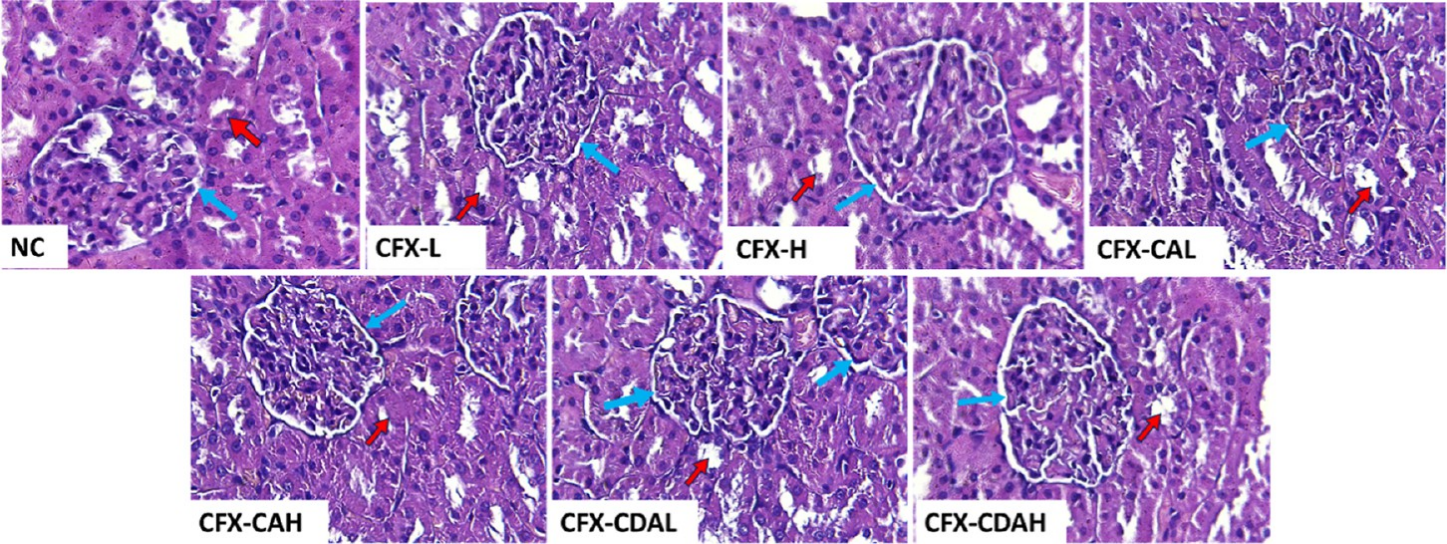
Histopathological section of the kidney of rat, which had been treated with different concentrations of CFX–CA and CFX–CDA. NC, negative control, CFX-L (ciprofloxacin-low dose), CFX-H (ciprofloxacin-high dose), CFX–CAL (CFX–CA-low dose), CFX–CAH (CFX–CA-high dose), CFX–CDAL (CFX–CDA-low dose), CFX–CDAH (CFX–CDA-high dose), CFX–CDAL shows renal corpuscles (blue arrows) having a clear capsular space with intact visceral and parietal layers and glomerular capillaries. Renal tubules (red arrows).

**Figure 12:**
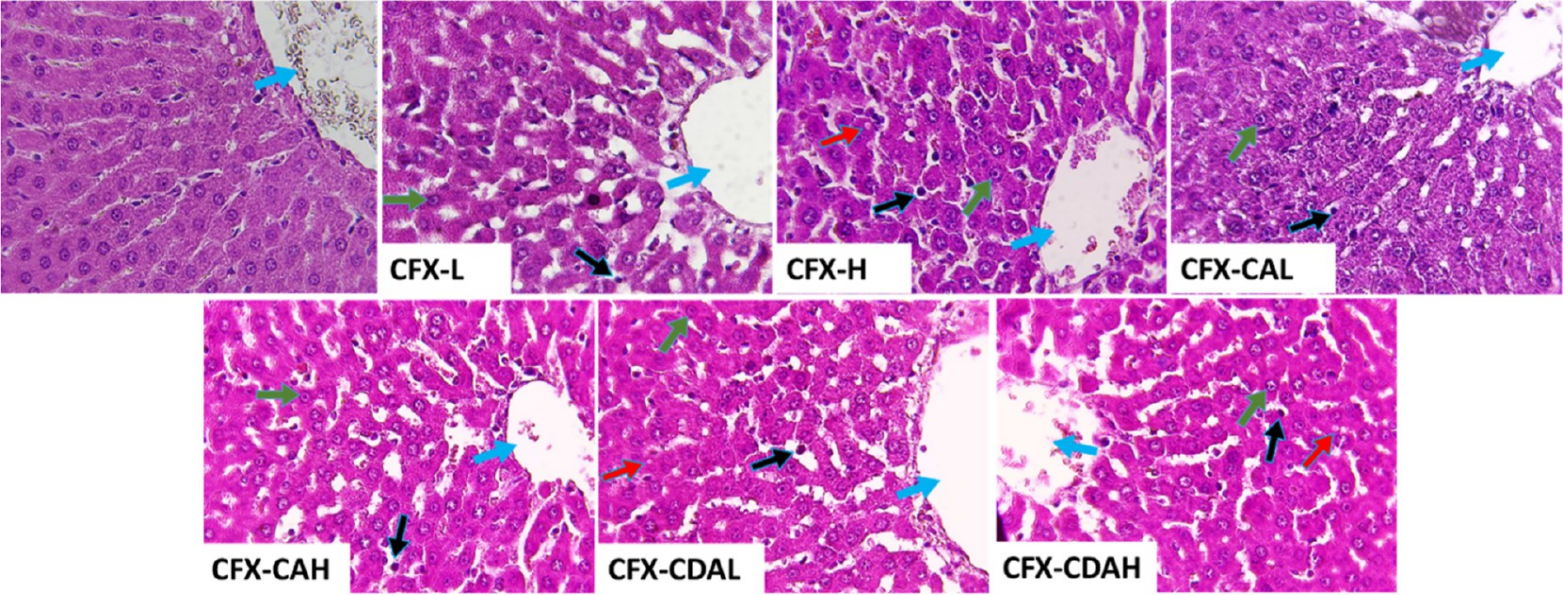
Histopathological section of the liver of rat, which had been treated with different concentrations of CFX–CA and CFX–CDA. NC, negative control, CFX-L (ciprofloxacin-low dose), CFX-H (ciprofloxacin-high dose), CFX–CAL (CFX–CA-low dose), CFX–CAH (CFX–CA-high dose), CFX–CDAL (CFX–CDA-low dose), CFX–CDAH (CFX–CDA-high dose), CFX–CDAL shows hepatic intracytoplasmic vacuole (red arrow), mild lymphocytic infiltration (black arrow), central vein and congestion central vein (blue arrow), and prominent nucleoli (green arrow) (H & E stain, 100x).

#### 3.3.1 Predictive Performance and Feature Importance in ML Models

##### 3.3.1.1 Model Screening and Performance Evaluation

To integrate the findings from solid-state characterization, solubility, aerodynamic performance, and in vivo toxicological results, ML models were used to determine the most influential formulation and process parameters affecting key performance attributes. An initial model selection screening (Figure 13) was conducted to evaluate multiple ML models. These models were assessed based on *R* ^2^ and RMSE to determine their predictive accuracy for each target variable. Three best-performing models were plotted, and the variability among them was expressed in terms of the violin length or distribution width. The violin plot shows the distribution of feature importance across models, while the error bar shows its variability with the standard deviation. Across targets, features like inlet temperature, molar ratio, weighted enthalpy of vaporization, and weighted solubility parameters consistently rank high across the top three models, indicating strong feature stability. These features appear repeatedly in the top positions for CPF, crystallinity, FPF, and solubility, suggesting that they play a key role regardless of the model choice. However, the predictive performance for CPF remained consistently low across all models compared to other targets, as confirmed by both *R* ^2^ and RMSE scores. To further validate this, additional diagnostics were performed (see Figure 16), including a learning curve and residual plot from 5-fold cross-validation for the Gradient Boosting model. These confirmed that CPF models showed signs of overfitting and limited generalizability and exhibited prediction clustering with high residual dispersion. This suggests that CPF may be influenced by additional experimental variables not captured in the current feature set.

**Figure 13:**
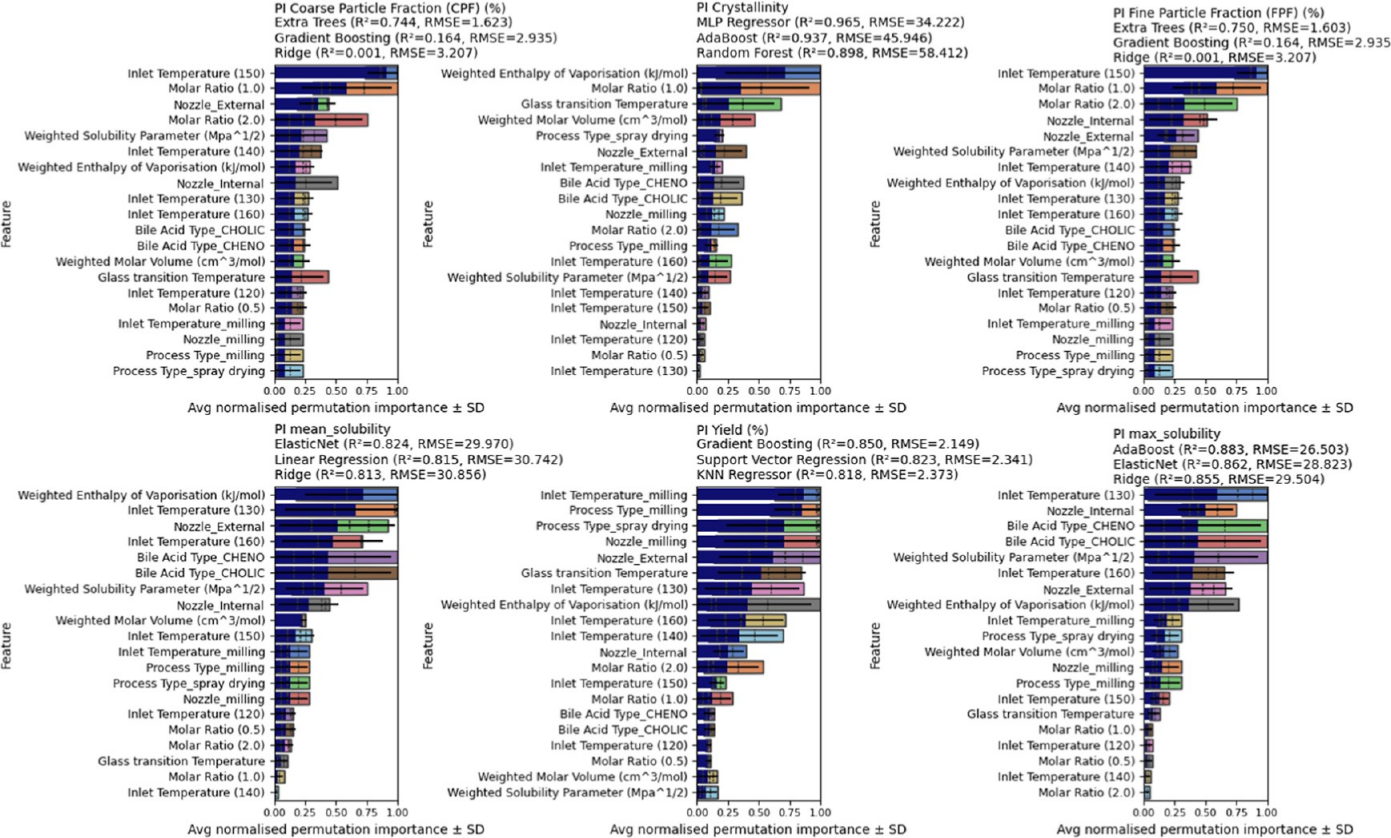
Comparative analysis of the top three ML models for predicting key formulation properties, highlighting model performance (*R* ^2^ and RMSE), feature importance rankings, and variability in predictive influence across different models.

##### 3.3.1.2 Selection of Best Models Based on Robustness

For each formulation target property, the best-performing model was selected from the top three candidates (Figure 14) based on the combination of statistical accuracy and generalization robustness, as revealed by diagnostic evaluations (see Supporting Information). While most of the top three models showed competitive *R* ^2^ and RMSE values, the final choice was not solely based on the highest *R* ^2^. Instead, model generalizability, residual distribution, and overfitting risk were carefully considered using metrics such as training vs validation performance divergence and residual spread.

**Figure 14:**
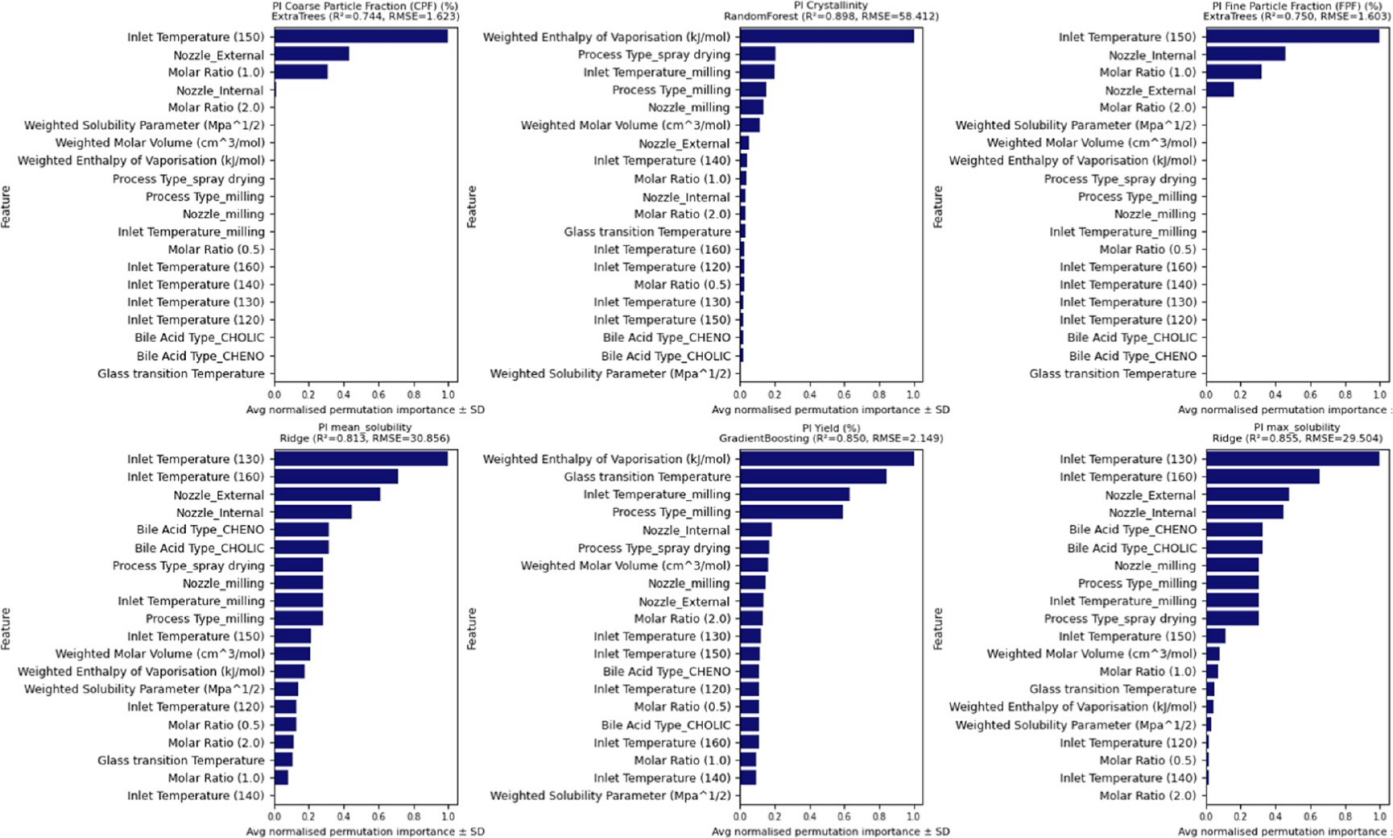
Permutation importance plots for crystallinity, dissolution, solubility, yield %, CFP %, and FPF %. Each subplot highlights the ranked significance of process parameters (e.g., inlet temperature and nozzle configuration) and material properties (e.g., bile acid type and molar ratio) in predicting the respective outcomes. Analysis is based on the best-performing ML model for each formulation property, highlighting optimal model performance (*R* ^2^ and RMSE), feature importance rankings, and variability in predictive influence within the selected model.

For example, Random Forest was selected for crystallinity despite other models (e.g., MLP) showing similar or slightly better *R* ^2^, due to its more balanced generalization across folds and lower residual dispersion. In the case of CPF (%) and FPF (%), Extra Trees offered consistent predictions and minimal overfitting, making them preferable over more complex learners that exhibited higher variance. For yield (%), Gradient Boosting was chosen due to its strong validation *R* ^2^, tight residual clustering, and smooth learning curve convergence. Similarly, Ridge Regression was selected for both mean and max solubility due to its excellent fit with low overfitting risk and near-perfect learning curve behavior, indicating model stability even with a limited data set.

##### 3.3.1.3 Feature Importance Analysis and Experimental Correlation

As can be seen in Figure 14, bile acid type was found to be the strongest predictor of solubility, reinforcing solubility parameter calculations and FTIR findings, where CDA exhibited stronger hydrogen bonding and better miscibility with CFX than CA. Similarly, inlet temperature, nozzle type, and molar ratio were the most significant predictors of aerodynamic performance, aligning with ACI results, which showed enhanced lung deposition efficiency at 150 °C. The ranking of *T*_g_ and enthalpy of vaporization as dominant predictors of crystallinity is consistent with DSC findings, confirming that amorphization is largely dependent on molecular cohesion.

The ML models also provided insights into how different formulation features influence microbiological and biological assays including MIC and toxicity evaluations. While MIC and toxicity studies were conducted in a solvent mixture where solubility differences would not be a limiting factor, the ML-predicted solubility trends correlated strongly with CFX release profiles, offering an indirect explanation for the differences in antimicrobial activity between CFX–CDA and CFX–CA formulations. The faster dissolution of CFX–CDA, which exhibited the highest solubility enhancement of up to 6-fold, potentially led to improved antimicrobial efficacy. This aligns with MIC results, where CFX–CDA formulations demonstrated a slightly stronger antibacterial activity with lower MIC. In contrast, CFX–CA formulations, which exhibited lower solubility and relatively weaker molecular interactions as predicted by ML, corresponded to reduced antimicrobial efficacy in MIC assays. The lower dissolution potential of CFX–CA aligns with the experimental solubility parameter calculations, which indicated weaker hydrogen bonding and miscibility with CFX. Although these findings do not directly impact MIC assays performed in solvent systems, they provide a mechanistic explanation for differences in the dissolution behavior, which may be relevant to drug bioavailability and efficacy in pulmonary conditions.

Toxicity studies provided additional insights into the relationship among solubility, dissolution kinetics, and cytotoxicity. Cell viability assays indicated that formulations with higher solubility and rapid release rates resulted in increased cytotoxicity, reinforcing the importance of a controlled drug release strategy. The ML models identified enthalpy of vaporization and *T*_g_ as the key predictors of crystallinity, which relate to drug stability and controlled release kinetics, thereby influencing toxicity outcomes. The findings suggest that formulations with higher amorphization may exhibit faster dissolution rates, which, if unregulated, could lead to cytotoxic effects due to rapid drug exposure. This observation is particularly relevant to inhalation formulations where maintaining controlled release kinetics is essential to achieving a balance between efficacy and safety.

CDA was confirmed as the superior coformer, providing enhanced solubility and amorphization stability compared to CA. Spray drying at 150 °C with an internal nozzle optimized fine particle formation, ensuring improved lung deposition, as confirmed by ACI data, which demonstrated increased FPF and reduced CPF for CFX–CDA formulations. A 1:1 CFX/CDA ratio provided the best balance between solubility enhancement, amorphization stability, antimicrobial activity, and inhalation efficiency, aligning with experimental and ML-derived predictions.

The findings demonstrate that bile acid selection, inlet temperature, and nozzle type are critical formulation and process parameters in predicting and explaining variations in inhalable dispersions. The three-fluid nozzle spray drying system, particularly at an inlet temperature of 150 °C, was found to be the most effective processing method for achieving an amorphous state while ensuring aerodynamic properties suitable for deep lung deposition. The combination of CDA as a stabilizing coformer and optimized spray-drying conditions resulted in formulations with improved solubility, amorphization, and antimicrobial efficacy.

##### 3.3.1.4 SHAP Interaction Analysis for Key Targets

To complement global permutation importance rankings, SHAP interaction plots were generated for three representative targets, solubility, crystallinity, and CPF, each with three key feature pairings (Figure 15). These plots elucidate how pairs of formulation or process variables interact to influence predictions, offering sample-level insights that permutation scores alone cannot capture. For CPF, inlet temperature showed a strong positive impact when paired with the external nozzle, while the internal nozzle yielded lower CPF values even at similar temperatures, highlighting the importance of process–device synergy. A second CPF plot showed that high molar ratios enhanced CPF only when the inlet temperature exceeded ∼140 °C, revealing a temperature threshold below which the molar ratio had limited effect. CPF was also modulated by the nozzle configuration and bile acid identity, where CDA-based formulations performed better with internal nozzles, pointing to physicochemical compatibility between droplet breakup dynamics and bile acid surface activity.

**Figure 15:**
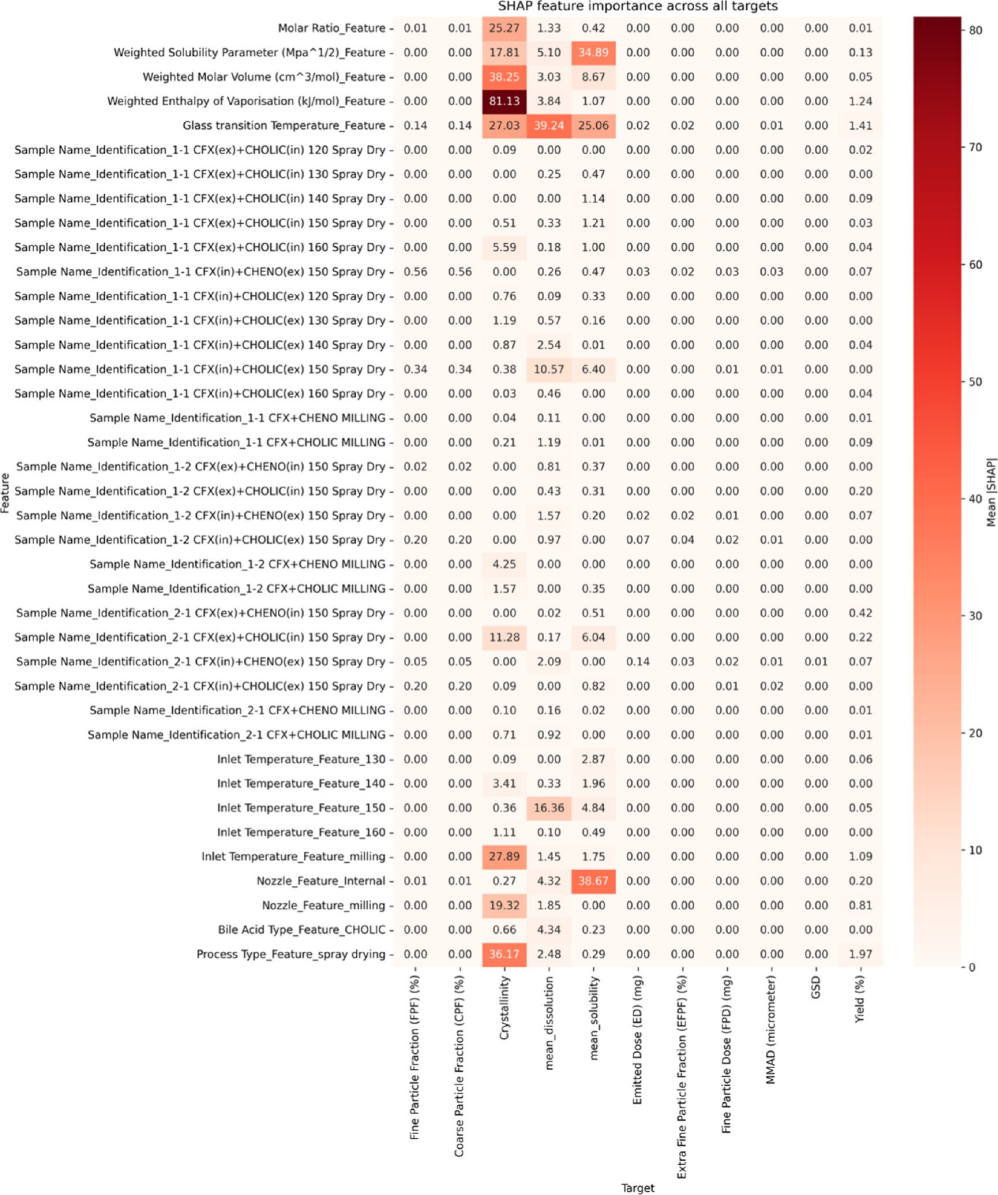
Heatmap showing the average SHAP values for top-ranked features across multiple predictive targets. Each cell represents the mean SHAP value for a given feature–target pair, quantifying the feature’s contribution to the model output. Darker shades indicate higher importance. Most sample identifier features show near-zero values, confirming they contributed little to predictions, whereas physicochemical and process variables (e.g., enthalpy of vaporization, *T*_g_, solubility parameter, inlet temperature, and nozzle type) consistently show strong influence across targets.

For crystallinity, SHAP revealed that the solubility parameter modulated the effect of the molar ratio: at higher solubility parameters, increasing the molar ratio reduced crystallinity more strongly, possibly due to enhanced miscibility disrupting crystal packing. The interplay between the enthalpy of vaporization and the bile acid type suggested that CDA-based systems required higher enthalpy of vaporization values to achieve lower crystallinity in line with the idea that more volatile systems aid amorphization but only when the bile acid structure allows. A further interaction between the enthalpy of vaporization and *T*_g_ showed that low *T*_g_ materials were more sensitive to changes in enthalpy of vaporization, with crystallinity decreasing steeply as the enthalpy of vaporization rose, consistent with glass-forming tendencies under energetic drying.

Solubility-related plots revealed that high enthalpy of vaporization only improved solubility when accompanied by a high solubility parameter, indicating that both volatility and compatibility were necessary to enhance the dissolution potential. Interestingly, enthalpy of vaporization’s influence was further amplified when *T*_g_ was low, suggesting that materials more readily mobilized at lower thermal transitions can take fuller advantage of high evaporation enthalpy. Lastly, the molar ratio–bile acid interaction showed that CDA-based systems responded better to increased molar ratios than CA-based ones, highlighting the compositional tuning needed for optimal solubilization.

##### 3.3.1.5 Diagnostic Validation and Generalizability

The reliability of the selected ML models was further assessed through diagnostic tools, as shown in Figure 16. The learning curves (top panel) plot *R* ^2^ against the training size for both the training (blue) and validation (orange) sets. Models predicting solubility and yield displayed smooth convergence at high *R* ^2^ values, indicating stable learning and good generalizability. These outcomes align with the experimental findings, where solubility and yield trends were consistent across formulations and strongly dependent on the bile acid type and processing conditions. In contrast, CPF and FPF exhibited a clear divergence between training and validation curves, a hallmark of overfitting. This matches the ACI experiments, which showed greater variability in deposition patterns, suggesting that additional aerosolization factors not captured in the feature set contribute to these end points. Crystallinity models showed partial convergence, reflecting the intermediate behavior consistent with DSC and XRPD, which revealed mixtures of amorphous and partially crystalline states depending on the composition and processing. The residual plots (bottom panel) provide complementary insights. Narrow, centered residuals in solubility and yield models confirm accurate predictions across the data set, whereas the broader scatter in CPF and crystallinity models indicates systematic error and missing explanatory features. The wide dispersion of CPF residuals in particular suggests that particle deposition behavior depends on variables not included in the current descriptors, such as capsule-emptying efficiency or device-specific airflow effects observed during ACI testing.

**Figure 16:**
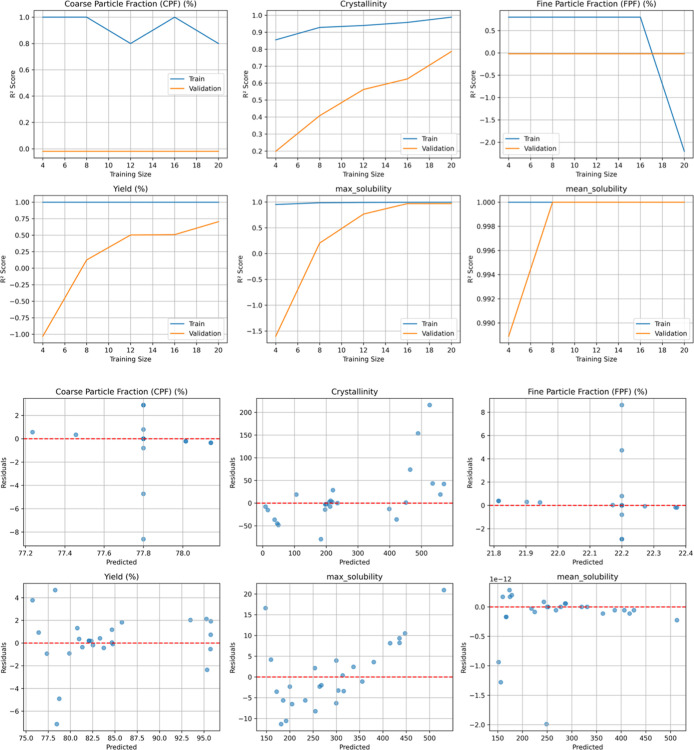
Diagnostic evaluations of the best-performing ML models for six formulation properties (CPF, crystallinity, FPF, yield, max_solubility, and mean_solubility). The top panel shows learning curves of *R* ^2^ score against training size for training (blue) and validation (orange) sets, illustrating model generalizability. High and converging *R* ^2^ values (e.g., solubility and yield) indicate a stable predictive behavior, whereas low or diverging *R* ^2^ values (e.g., CPF and FPF) suggest overfitting and missing explanatory variables. The bottom panel shows residual plots (actual – predicted vs predicted values), with the red dashed line indicating ideal prediction. Narrow, centered residuals (e.g., mean_solubility) suggest accurate predictions, while wider scatter (e.g., crystallinity and CPF) reflects unaccounted variability. Note: residual axes are scaled individually to highlight dispersion patterns for each property.

Together, these diagnostics highlight where the models were robust and where they were limited. Importantly, interpretability tools such as permutation importance and SHAP consistently identified mechanistic relationships—for example, inlet temperature and molar ratio driving solubility and crystallinity—that mirrored experimental evidence from dissolution, DSC, and XRPD. Thus, while not all targets could be predicted reliably (e.g., CPF and FPF), the convergence of ML-derived and experimental insights supports the internal consistency of the findings. Although the data set size inevitably limits broader generalizability, the combined approach establishes a framework for rational formulation design, to be strengthened through future studies with larger data sets.

## 4 Conclusions

This study demonstrates the potential of bile acids (particularly CDA) as effective coformers for enhancing the solubility, stability, and pulmonary delivery of CFX via DPIs. Both CA and CDA substantially reduced drug crystallinity, as confirmed by DSC and XRPD, and improved solubility by up to 6-fold compared to crystalline CFX. In addition to their solubilizing effect, bile acids showed potential as bioactive agents capable of disrupting bacterial membranes, suggesting a dual-function strategy for treating resistant lung infections. While CA exhibits closer Hildebrand solubility parameters to CFX implying better thermodynamic compatibility, experimental results from FTIR, *T*_g_, and aerosol deposition studies consistently indicated that CDA formed stronger specific interactions with CFX. This highlights a key limitation of relying solely on solubility parameters, which do not account for directional hydrogen bonding or molecular geometry. ML complemented the experimental data by identifying critical formulation and process variables such as inlet temperature, nozzle configuration, and molar ratio that govern key quality attributes, including solubility, dissolution, crystallinity, and FPF. By integrating ML-derived mechanistic insights with experimental validation, this approach provides a robust framework for rational DPI formulation design, potentially outperforming conventional carrier-based systems in achieving targeted lung delivery and therapeutic efficacy. However, our in vivo findings also indicated that CDA formulations produced mild, dose-dependent hepatic effects (AST elevation at low dose and ALP increase at high dose), whereas CA formulations showed a safety profile broadly comparable to that of CFX. These hepatic signals highlight a translational limitation for CDA that warrants careful evaluation in longer-term studies.

## Supplementary Material









## Notes

Detailed solubility parameter calculations for CFX and bile acids, as well as additional model performance data (XLSX)
Best model comparison results (XLSX)
Calculations of solubility parameters (PDF)
Additional data on the lipid profile, RBC and hematological parameters, and white blood cell and platelet counts from in vivo toxicological studies and ML analyses, including the learning curve and residual plots for the Gradient Boosting model (PDF)
