# Supplementary material for: Machine Learning-Guided Prediction of Formulation Performance in Inhalable Ciprofloxacin–Bile Acid Dispersions with Antimicrobial and Toxicity Evaluation
Source: Mol Pharm. 2025 Oct 11;22(11):6681–702. doi: 10.1021/acs.molpharmaceut.5c00663 (PMC12587441; doi:10.1021/acs.molpharmaceut.5c00663)
Supplement: Supplementary file 3 [file mp5c00663_si_003.pdf]

Supplementary section- calculations of solubility parameters

Cholic acid - group contributions and solubility parameter calculation

Group	Quantity	Per Group Energy (cal/mole)	Total Energy (cal/mole)
CH_3 (methyl)	3	1125	3375
CH_2 (methylene)	6	1180	7080
CH (single-bonded)	4	820	3280
OH (hydroxyl)	3	8970	26910
COOH (carboxyl)	1	6600	6600
Total	-	-	47245
Molar volume (ChemSpider)	-	-	344.8 cm^3/mole
Hildebrand solubility parameter (δ) $(cal/cm^3)^{1/2}$	-	-	11.71
Hildebrand solubility parameter (δ) ($MPa^{1/2}$)	-	-	23.94

Chenodeoxycholic acid - group contributions and solubility parameter calculation

Group	Quantity	Per Group Energy (cal/mole)	Total Energy (cal/mole)
CH_3 (methyl)	3	1125	3375
CH_2 (methylene)	6	1180	7080
CH (single-bonded)	4	820	3280
OH (hydroxyl)	2	8970	17940
COOH (carboxyl)	1	6600	6600
Total	-	-	38275
Molar volume (ChemSpider)	-	-	347.9 cm^3/mole
Hildebrand solubility parameter (δ) $(cal/cm^3)^{1/2}$	-	-	10.49
Hildebrand solubility parameter (δ) ($MPa^{1/2}$)	-	-	21.46

ciprofloxacin - group contributions and solubility parameter calculation

Group	Quantity	Per Group Energy (cal/mole)	Total Energy (cal/mole)
CH_3 (methyl)	2	1125	2250
CH_2 (methylene)	2	1180	2360
C= (aromatic & carbonyls)	8	1030	8240
OH (hydroxyl)	1	800	800
F (fluorine)	1	6800	6800
N (tertiary amine & aromatic N)	2	2000	4000
CO (ketone)	1	4300	4300
COOH (carboxyl)	1	4800	4800
Total	-	-	32550
Molar volume (ChemSpider)	-	-	226.8 cm^3/mole
Hildebrand solubility parameter (δ) $(cal/cm^3)^{1/2}$	-	-	12.16
Hildebrand solubility parameter (δ) ($MPa^{1/2}$)	-	-	24.88
