# Supplementary material for: Machine Learning-Guided Prediction of Formulation Performance in Inhalable Ciprofloxacin–Bile Acid Dispersions with Antimicrobial and Toxicity Evaluation
Source: Mol Pharm. 2025 Oct 11;22(11):6681–702. doi: 10.1021/acs.molpharmaceut.5c00663 (PMC12587441; doi:10.1021/acs.molpharmaceut.5c00663)
Supplement: Supplementary file 4 [file mp5c00663_si_004.pdf]

Machine learning-guided prediction of formulation performance in inhalable ciprofloxacin–bile acid dispersions with antimicrobial and toxicity evaluation

Tareq Zeyad Bahjat[1], Twana Mohammed M. Ways[2], Sadat Abdulla Aziz[3,4], Aram Ismael Ibrahim[2], Deon Danto[1], Veneece Ghattas[1], Goran Mohammed Raouf[5], Glyn Barrett[6], Dana Khdr Sabir[7], Pyman Mohamed Mohamedsalih[7], Hisham Al-Obaidi[1*]

[1] School of Pharmacy, University of Reading, Reading, RG6 6AD, UK
[2]Department of Pharmaceutics, College of Pharmacy, University of Sulaimani, Sulaymaniyah, 46001, Kurdistan Region, Iraq.

[3]Department of Anaesthesia, Cihan University-Sulaimaniya, College of health sciences, Sulaymaniyah, *Kurdistan Region, Iraq*

[4]Department of Basic Sciences, *College of Vet. Medicine, University of Sulaimani,* Sulaymaniyah, 46001, Kurdistan Region, Iraq

[5]Basic Medical Sciences, College of Medicine, University of Sulaimani, Sulaymaniyah, 46001, Kurdistan Region, Iraq.

[6]School of Biological Sciences, University of Reading, Reading RG6 6AD, UK

[7]Department of Medical Laboratory Sciences, College of Science, Charmo University, 46023 Chamchamal, Kurdistan Region, Iraq

Author Contributions:

TZB: conceptualisation, investigation, methodology, writing – original draft, review & editing.TMW, SA: *in vivo* investigation, methodology, writing – original draft, review, editing & data analysis; AII, in vivo investigation, GMR; Histopathological investigation, GB: methodology, investigation, writing – original draft, review & editing; HAO: conceptualisation, investigation, methodology, project administration, writing – original draft, review & editing. DKS & PMM: MIC test.

*Corresponding author: h.al-obaidi@reading.ac.uk

Keywords: Ciprofloxacin; Dry powder inhaler (DPI); Solid dispersions; Bile acids; Spray drying; Lung deposition; Antimicrobial activity; Machine learning

Table S1: Lipid profile of the treated rats with different concentrations of CFX-CA and CFX-CDA.

Tests / Treatments	Triglyceride (TG) (mg/dL)	Cholesterol (mg/dL)	Low-Density Lipoprotein (LDL) (mg/dL)	High-Density Lipoprotein (HDL) (mg/dL)	Very low-Density Lipoprotein (VLDL)(mg/dL)
**NC**	75.45±4.35^{A}	108.05±2.6^{C}	26.5±2.4^{C}	71.0±2.2^{B}	15.0±1.0^{A}
**CFX-L**	69.3±16.32^{A}	93.53±23.2^{BC}	22.93±5.87^{BC}	62.87±15.75^{B}	14.0±3.0^{A}
**CFX-H**	72.33±20.02^{A}	79.13±3.2^{AB}	16.57±3.16^{AB}	54.9±2.95^{AB}	14.33±3.79^{A}
**CFX-CAL**	64.07±16.65^{A}	95.1±9.4^{BC}	22.9±3.64^{BC}	60.8±7.18^{B}	13.0±3.61^{A}
**CFX-CAH**	104.13±44.79^{A}	92.5±9.4^{BC}	23.2±0.9^{BC}	60.47±14.81^{B}	20.67±9.29^{A}
**CFX-CDAL**	98.1±18.63^{A}	70.5±11.4^{A}	15.77±4.46^{A}	40.67±10.82^{A}	19.33±3.79^{A}
**CFX-CDAH**	65.17±7.28^{A}	79.0±7.2^{AB}	18.37±1.59^{AB}	54.07±2.97^{AB}	13.0±1.73^{A}

*NC; Negative control, CFX-L(ciprofloxacin-Low dose), CFX-H(ciprofloxacin-high dose), CFX-CAL; CFX-CA-low dose, CFX-CAH; CFX-CA -high dose, CFX-CDAL; CFX-CDA-low dose, CFX-CDAH; CFX-CDA -high dose. Values are means± standard deviations for N=3. Statistical analysis was by one-way ANOVA. Superscript letters indicate results of post-hoc multiple comparisons ($p < 0.05$). Groups with the same letter are not significantly different. Groups with no letters in common (e.g., A vs C) differ significantly. Mixed labels (e.g., AB, BC) indicate overlap, meaning that group is not significantly different from either of the letter groups it shares, but the two letter groups themselves (e.g., A vs C) may still differ.

Table S2: Red blood cells values of the treated rats with blank and different concentrations of CFX-CA and CFX-CDA.

Tests / Treatments	Red Blood Cell (RBC) ($10^6/\mu L$)	Haemoglobin (Hgb) (g/dL)	Haematocrit% (HCT)	Mean Corpuscular Volume (MCV) (μmol)	Mean Corpuscular Haemoglobin (MCH) (Pg)	Mean Corpuscular Haemoglobin Concentration (MCHC) (g/dL)	RDW C%	RDWS (μm)
**NC**	6.3±0.6^{A}	13.3±0.5^{A}	33.6±4.4^{A}	61.9±4.9^{A}	21.4±1.4^{A}	34.5±0.5^{B}	17.8±0.15^{A}	44.5±3.7^{AB}
**CFX-L**	7.14±0.6^{B}	14.3±1.6^{AB}	42.3±4.2^{B}	59.1±2.5^{A}	20.0±0.7^{A}	30.5±4.9^{A}	17.5±2.9^{A}	42.8±9.5^{AB}
**CFX-H**	7.15±0.5^{B}	14.2±0.9^{AB}	43.0±2.4^{B}	60.2±2.5^{A}	19.9±0.4^{A}	33.1±0.7^{AB}	19.4±4.5^{A}	46.8±10.7^{AB}
**CFX-CAL**	7.05±0.3^{AB}	14.0±0.1^{AB}	42.5±4.1^{B}	58.0±4.1^{A}	19.9±0.8^{A}	33.9±1.9^{AB}	17.4±2.4^{A}	40.6±6.0^{AB}
**CFX-CAH**	6.9±0.5^{AB}	14.3±0.5^{AB}	41.2±1.2^{B}	59.2±2.4^{A}	20.5±0.7^{A}	34.6±0.3^{B}	16.4±0.1^{A}	38.6±1.9^{A}
**CFX-CDAL**	7.08±0.1^{AB}	15.3±0.6^{B}	44.3±2.0^{B}	62.4±4.1^{A}	21.5±1.3^{A}	34.4±0.5^{AB}	20.6±2.7^{AB}	52.1±3.8^{BC}
**CFX-CDAH**	6.3±0.2^{AB}	13.2±0.1^{A}	39.1±0.5^{B}	60.9±0.7^{A}	20.6±0.7^{A}	33.1±0.5^{AB}	23.9±0.5^{B}	60.4±0.6^{C}

*NC; Negative control, CFX-L(ciprofloxacin-Low dose), CFX-H(ciprofloxacin-high dose), CFX-CAL; CFX-CA-low dose, CFX-CAH; CFX-CA -high dose, CFX-CDAL; CFX-CDA-low dose, CFX-CDAH; CFX-CDA -high dose.

* Superscripts on the right of the means indicate statistical differences ($P<0.05$) between the groups. Values are means± standard deviations for N=3. Statistical analysis was by one-way ANOVA. Superscript letters indicate results of post-hoc multiple comparisons ($p < 0.05$). Groups with the same letter are not significantly different. Groups with no letters in common (e.g., A vs C) differ significantly. Mixed labels (e.g., AB, BC) indicate overlap, meaning that group is not significantly different from either of the letter groups it shares, but the two letter groups themselves (e.g., A vs C) may still differ.

Table S3: Total and differential white blood cells (WBCs) count, and platelets (PLT) values of the treated rats with blank and different concentrations of CFX-CA and CFX-CDA.

Treatments	White Blood Cells (WBC) (103 /μL)	Neutrophil%	Lymphocytes%	Monocytes%	Eosinophils%	Basophils%	Platelets (PLT) (103/μL)
**NC**	7.51±0.9 ^{AB}	35.4±0.2 ^{BC}	57.2±3.4 ^{ABC}	6.7±3.2 ^{ABC}	0.01±0.01 ^{A}	0.48±0.3 ^{A}	624±38 ^{A}
**CFX-L**	7.05±1.52 ^{AB}	35.8±7.4 ^{BC}	58.1±5.1 ^{BC}	6.6±1.2 ^{ABC}	0.1±0.2 ^{AB}	0.46±0.15 ^{A}	603±153 ^{A}
**CFX-H**	7.41±1.1 ^{AB}	39.6±6.1 ^{C}	47.5±3.4 ^{A}	8.7±1.8 ^{BCD}	0.07±0.11 ^{AB}	0.4±0.1 ^{A}	563±19 ^{A}
**CFX-CAL**	6.81±1.6 ^{AB}	34.9±1.2 ^{BC}	55.8±11.1 ^{ABC}	4.9±0.8 ^{A}	1.8±1.5 ^{C}	0.36±0.11 ^{A}	596±144 ^{A}
**CFX-CAH**	5.47±1.8^{A}	30.6±2.9 ^{AB}	61.2±3.1 ^{BC}	6.2±1.1 ^{AB}	1.3±0.9 ^{BC}	0.34±0.15 ^{A}	564±25 ^{A}
**CFX-CDAL**	11.21±1.3 ^{C}	23.9±3.5 ^{A}	64.6±4.7 ^{C}	10.3±1.2 ^{D}	0.6±0.05 ^{AB}	0.3±0.1 ^{A}	674±79 ^{A}
**CFX-CDAH**	8.31±0.37 ^{B}	38.3±0.4 ^{BC}	51.2±0.2 ^{AB}	9.4±0.25 ^{CD}	0.20±0.01 ^{AB}	0.24±0.06 ^{A}	514±10 ^{A}

*NC; Negative control, CFX-L(ciprofloxacin-Low dose), CFX-H(ciprofloxacin-high dose), CFX-CAL; CFX-CA-low dose, CFX-CAH; CFX-CA-high dose, CFX-CDAL; CFX-CDA-low dose, CFX-CDAH; CFX-CDA-high dose.

* Superscripts on the right of the means indicate statistical differences (P<0.05) between the groups. Values are means± standard deviations for N=3. Statistical analysis was by one-way ANOVA. Superscript letters indicate results of post-hoc multiple comparisons (p < 0.05). Groups with the same letter are not significantly different. Groups with no letters in common (e.g., A vs C) differ significantly. Mixed labels (e.g., AB, BC) indicate overlap, meaning that group is not significantly different from either of the letter groups it shares, but the two letter groups themselves (e.g., A vs C) may still differ.

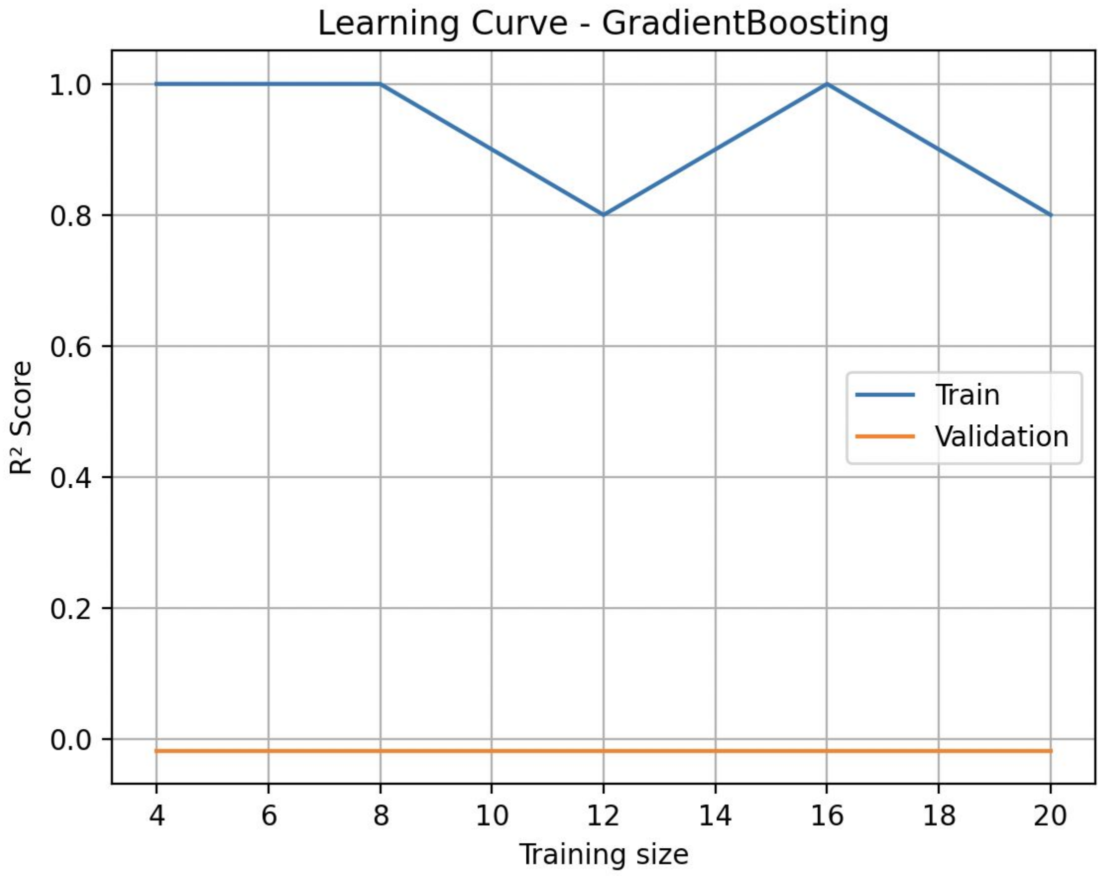

Figure S1. Learning curve of Gradient Boosting model for CPF prediction. Train score remains near 1.0 across all training sizes, while validation score remains flat at 0, indicating overfitting and no generalisation.

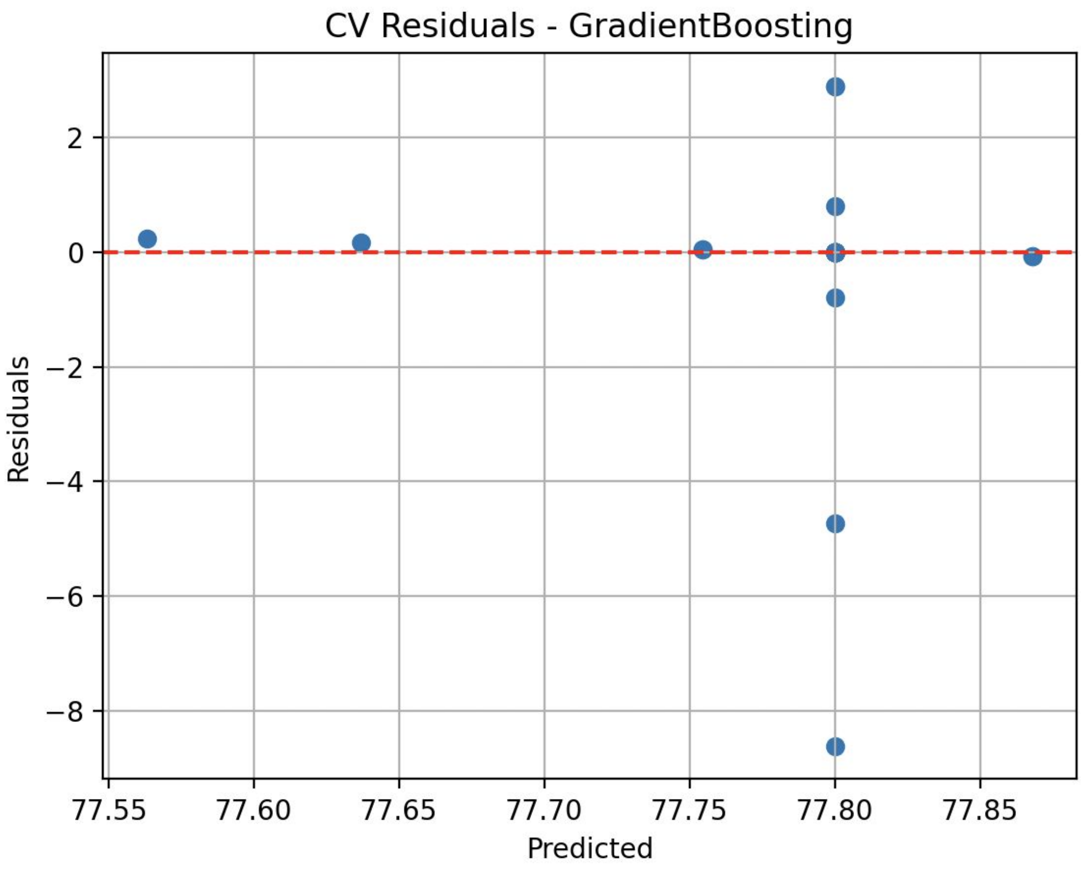

Figure S2. Cross-validated residuals of Gradient Boosting model for CPF. Predictions are narrowly distributed around a central value (~77.8), while residuals vary widely, showing instability and lack of predictive capacity.
